# Viral Infection of the Central Nervous System Exacerbates Interleukin-10 Receptor Deficiency-Mediated Colitis in SJL Mice

**DOI:** 10.1371/journal.pone.0161883

**Published:** 2016-09-09

**Authors:** Ann-Kathrin Uhde, Vanessa Herder, Muhammad Akram Khan, Malgorzata Ciurkiewicz, Dirk Schaudien, René Teich, Stefan Floess, Wolfgang Baumgärtner, Jochen Huehn, Andreas Beineke

**Affiliations:** 1 Department of Pathology, University of Veterinary Medicine Hannover, Hannover, Germany; 2 Center for Systems Neuroscience, Hannover, Germany; 3 Department of Pathobiology, Faculty of Veterinary & Animal Sciences, PMAS—Arid Agriculture University, Rawalpindi, Pakistan; 4 Fraunhofer—Institute for Toxicology and Experimental Medicine ITEM, Hannover, Germany; 5 Experimental Immunology, Helmholtz Centre for Infection Research, Braunschweig, Germany; Creighton University, UNITED STATES

## Abstract

Theiler´s murine encephalomyelitis virus (TMEV)-infection is a widely used animal model for studying demyelinating disorders, including multiple sclerosis (MS). The immunosuppressive cytokine Interleukin (IL)-10 counteracts hyperactive immune responses and critically controls immune homeostasis in infectious and autoimmune disorders. In order to investigate the effect of signaling via Interleukin-10 receptor (IL-10R) in infectious neurological diseases, TMEV-infected SJL mice were treated with IL-10R blocking antibody (Ab) in the acute and chronic phase of the disease. The findings demonstrate that (i) Ab-mediated IL-10 neutralization leads to progressive colitis with a reduction in Foxp3^+^ regulatory T cells and increased numbers of CD8^+^CD44^+^ memory T cells as well as activated CD4^+^CD69^+^ and CD8^+^CD69^+^ T cells in uninfected mice. (ii) Concurrent acute TMEV-infection worsened enteric disease-mediated by IL-10R neutralization. Virus-triggered effects were associated with an enhanced activation of CD4^+^ T helper cells and CD8^+^ cytotoxic T lymphocytes and augmented cytokine expression. By contrast, (iii) IL-10R neutralization during chronic TMEV-infection was not associated with enhanced peripheral immunopathology but an increased CD3^+^ T cell influx in the spinal cord. IL-10R neutralization causes a breakdown in peripheral immune tolerance in genetically predisposed mice, which leads to immune-mediated colitis, resembling inflammatory bowel disease. Hyperactive immune state following IL-10R blockade is enhanced by central nervous system-restricted viral infection in a disease phase-dependent manner.

## Introduction

Theiler´s murine encephalomyelitis virus (TMEV)-infection is a widely used animal model for studying demyelinating disorders, including human multiple sclerosis (MS). Following intracerebral infection, genetically susceptible mouse strains, such as SJL mice develop viral persistence with delayed-type hypersensitivity and myelin-specific autoimmunity with spinal cord myelin loss, resembling chronic progressive MS lesions [1–6]. In contrast, resistant C57BL/6 mice eliminate the virus from the central nervous system (CNS) by specific cellular immunity, including effector CD8^+^ cytotoxic T lymphocytes (CTL) responses during the acute infection phase [7].

Interleukin (IL)-10 is an anti-inflammatory cytokine secreted by a variety of cell types. The main functions of IL-10 include down-regulation of pro-inflammatory cytokine expression, reduction of antigen (Ag) presentation and reduced T cell activation [8–14]. Ligation of the interleukin-10 receptor (IL-10R) leads to phosphorylation and translocation of *signal transducer and activator of transcription 3* (STAT3) molecules, promoting the transcription of *suppressor of cytokine signaling 3* (SOCS3) leading to profound immune inhibitory effects [15, 16]. Thus, IL-10 counteracts hyperactive immune responses and critically controls immune homeostasis [17, 18]. In autoimmune CNS disorders, such as experimental autoimmune encephalomyelitis (EAE), IL-10 exerts protective effects by reducing T cell-mediated tissue damage [19]. The critical role of IL-10 in immune-mediated disorders is also demonstrated in human inflammatory bowel disease (IBD), a chronic, relapsing, idiopathic inflammation of the intestinal tract. Here, IL-10 signaling defects cause a particularly early onset of IBD and loss-of-function mutations affecting IL-10R contribute to the development of very early-onset-IBD, a serious enteric disease in children [20–25]. Moreover, genetic deficiency of either IL-10 or IL-10R in transgenic mice leads to a breakdown in immune tolerance and immune mediated colitis, representing a well-established murine model for IBD [26–28].

IL-10 also influences the disease outcome in several persistent viral infections [29–33]. For instance, the cytokine contributes to T cell exhaustion and persistence of lymphocytic choriomeningitis virus (LCMV)-infection in C57BL/6 mice, which can be circumvented by treatment with IL-10R blocking antibody (Ab) [29, 32]. Similarly, genetic and Ab-mediated blockade of IL-10 signaling decreases the mortality rate and brain virus load in murine West Nile Virus (WNV)-infection [31]. By contrast, IL-10 knockout mice infected with a neurotropic strain of mouse hepatitis virus exhibited overwhelming morbidity and increased mortality without affecting virus clearance [33]. In addition, IL-10 produced by CD8^+^ regulatory T cells (Treg) alleviates acute encephalitis in mouse hepatitis virus-infected mice, suggestive of IL-10-dependent mechanisms to reduce CNS immunopathology [34]. Thus, in contrast to primary beneficial effects of IL-10 in autoimmune disorders, an ambivalent function of IL-10 has to be considered in infectious CNS diseases, contributing to insufficient protective (e.g. antiviral) immunity on the one hand, but limiting immunopathology on the other [35]. Referring to this, in Theiler’s murine encephalomyelitis (TME) an enhanced expression of IL-10 has been measured in the brain of SJL mice. Nevertheless, the functional relevance of inhibitory cytokines and effects of pharmacological modulation of the IL-10 pathway remains largely undetermined in this MS model [36].

The aim of the present study was to gain insights into IL-10-mediated immune regulation in SJL mice which are prone to developing a variety of immune-mediated disorders, including TMEV-induced demyelination [37–43]. In order to determine disease-phase specific effects of CNS infection upon systemic immunopathology, IL10R deficiency was induced by administration of cytokine receptor blocking antibodies during the acute and chronic demyelinating TME phase, respectively. In addition, the potential impact of systemic IL-10R deficiency upon neuropathology in an infectious MS model was investigated. Results illustrate the ability of Ab-mediated IL-10R neutralization to cause an immune enhancement and progressive colitis in SJL mice, which are aggravated by concurrent TMEV-infection in a disease phase-dependent manner.

## Materials and Methods

### Mice and experimental design

**Fig 1:**
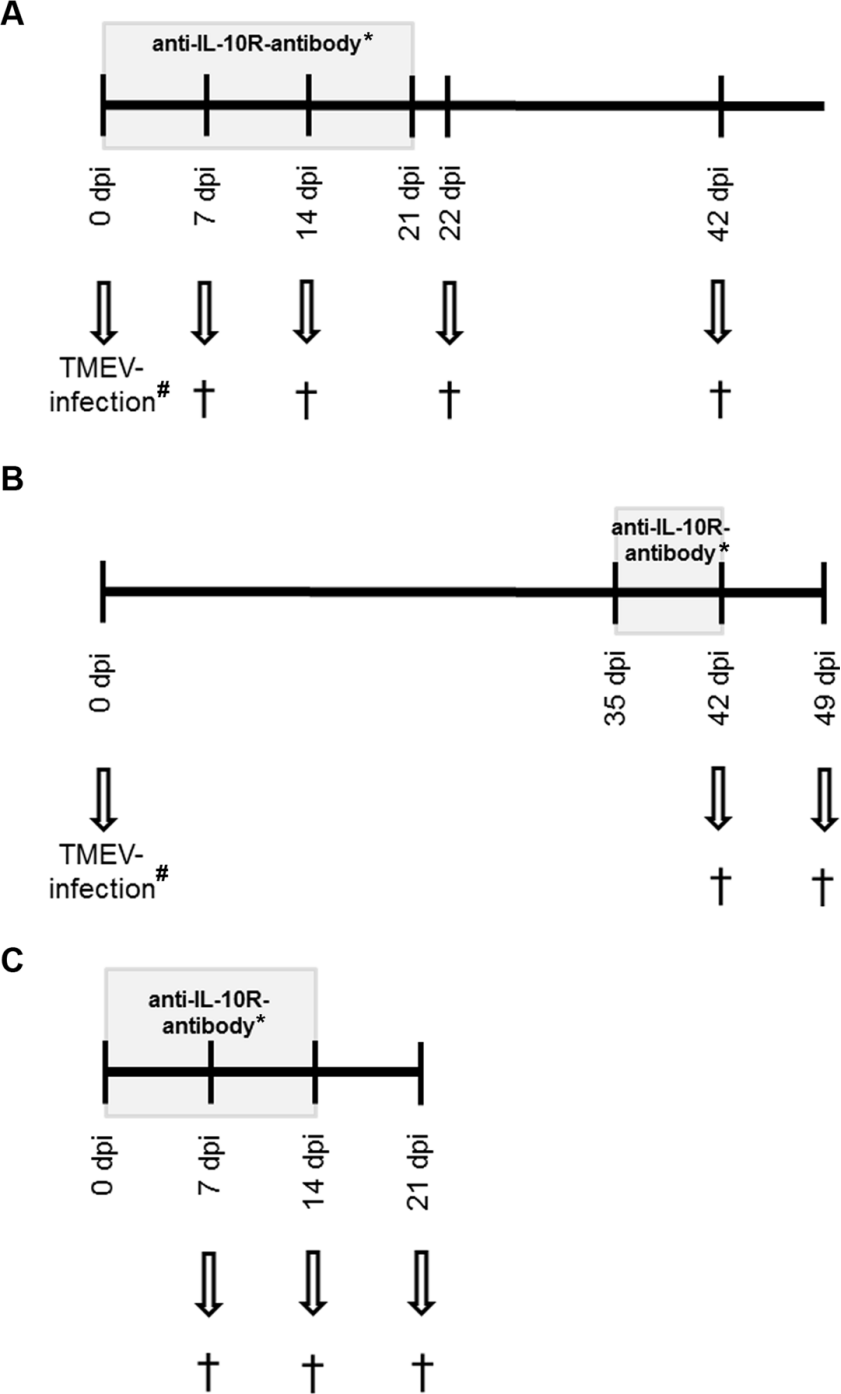
Experimental design. (A) *Experiment I*: animals infected with Theiler’s murine encephalomyelitis virus (TMEV) received IL-10 receptor blocking antibodies (IL-10R Ab) once weekly at day 0, 7, 14 and 21 of the experiment (grey box). Necropsy (†) was performed in groups of five animals at 7, 14, 22 and 42 days post infection (dpi). For negative controls a second group received IgG1-specific isotype control instead of IL-10R Ab (*) and a third group was mock-infected only (#) (B) *Experiment II*: TMEV-infected animals received IL-10R Ab once weekly at 35 and 42 dpi (grey box). Necropsy (†) was performed in groups of five animals at 42 and 49 dpi. For negative controls, a second group received IgG1-specific isotype control instead of IL-10R Ab (*) and a third group was mock-infected only (#) (C) *Experiment III*: animals received IL-10R Ab once weekly at day 0, 7 and 14 (grey box). Necropsy (†) was performed in groups of five animals at 7, 14 and 21 days after the first Ab application. For negative control a second group received IgG1-specific isotype control instead of IL-10R Ab (*).

Four-week-old, female, specific pathogen free SJL/JCrHsd mice were purchased from Harlan Winkelmann (Borchen, Germany) and group housed in isolated ventilated cages (Tecniplast, Hohenpeißenberg, Germany) with a 12-hour light/12-hour dark cycle. Animals had free access to tap water and a standard rodent diet (R/M-H, Ssniff Spezialdiäten GmbH, Soest, Germany) and were assigned randomly to three experiments: experiment I examined the effect of IL-10R blockade under acute infectious conditions before the onset of demyelination [44, 45]. Animals received a) rat anti-mouse IL10R antibody (Ab) and TMEV-infection (group “IL-10R↓_early_/TMEV”), b) immunoglobulin G1 (IgG1)-specific isotype control and TMEV-infection (group “isotype_early_/TMEV”) or c) rat anti-mouse IL10R Ab and mock-infection (group “IL-10R↓_early_/mock”), respectively. For comparison, experiment II included also three animal groups (groups “IL-10R↓_late_/TMEV”, “isotype_late_/TMEV”, and “IL-10R↓_late_/mock”) but IL10R Ab and isotype controls were administered at later time points to investigate IL-10R blockade during chronic TMEV-infection after the onset of demyelination [44, 45]. Experiment III was performed to investigate IL-10R signaling effects under non-infectious conditions and consisted of non-infected SJL mice which were treated with anti-IL-10R Ab (group “IL-10R↓”) or rat IgG1 isotype control (group “isotype”) only. Days of antibody application are described below (*in vivo* monoclonal antibody treatment). Each subgroup included five animals. The study design is illustrated in Fig 1.

All experiments were conducted in accordance with German law for animal protection and with the European Communities Council Directive for the protection of animals used for experimental purposes. Approvement and authorization of the animal experiment was given by the local authorities (Niedersächsisches Landesamt für Verbraucherschutz und Lebensmittelsicherheit (LAVES), Oldenburg, Germany, permission number: 33.12-42502-04-13/1138).

### In vivo monoclonal antibody treatment

Animals of experiment I were intraperitoneally injected with 250 μg rat anti-mouse IL-10R Ab (groups “IL-10R↓_early_/TMEV” and “IL-10R↓_early_/mock”; clone: 1B1.3A, BioXCell, West Lebanon, USA) for IL-10R blockade or rat immunoglobulin G1 (IgG1)-specific isotype control (group “isotype_early_/TMEV”; BioXCell, West Lebanon, USA) as described [31]. Animals were treated weekly at 0, 7, 14, and 21 dpi. In experiment II, IL-10R Ab (groups “IL-10R↓_late_/TMEV” and “IL-10R↓_late_/mock) and isotype control (group “isotype_late_/TMEV”) were administered at 35 and 42 dpi. In experiment III, mice received IL-10R Ab (group “IL-10R↓”) and isotype control (group “isotype”) at day 0, 7, and 14.

### Virus infection

At the age of five weeks (0 days post infection [dpi]) animals of experiment I and II) were intracerebrally inoculated with the BeAn-strain of TMEV or received mock-infection as described previously [46]. Briefly, intracerebral inoculation was carried out under general anesthesia with medetomidine (0.5 mg/kg, i.m., Domitor, Pfizer, Karlsruhe, Germany) and ketamine (100 mg/kg, intraperitoneal (i.p.), Ketamine 10 %, WDT eG, Garbsen, Germany). 1.63x10^6^ plaque-forming units of the virus resolved in 20 μl Dulbecco´s modified Eagle´s medium (PAA Laboratories, Cölbe, Germany) with 2 % fetal calf serum and 50 μg/kg gentamicin (Sigma-Aldrich Chemie GmbH, Taufkirchen, Germany) were injected into the right cerebral hemisphere. Mock-infected animals received 20 μl of the vehicle only intracerebrally.

### Clinical examination

During all experiments, weekly clinical scoring and RotaRod^®^ performance tests for quantifying motor coordination deficits were performed by two examiners (AU, AB) in a non-blinded manner as previously described [47–50]. Briefly, each animal was scored once weekly for the following clinical parameters: a) general appearance (0 = normal appearance; 1 = mild change [shaggy and scruffy hair]; 2 = moderate change [scruffy appearance and hunched back]; 3 = severe changes [unkempt appearance, urinary incontinence]), b) behavior and activity (0 = no change, 1 = mild change [mildly reduced spontaneous movement]; 2 = moderate change [moderately reduced spontaneous activity with unchanged induced activity]; 3 = severe change [no spontaneous movement, reduced induced activity]), and c) gait (0 = normal gait; 1 = mild change [mild ataxia with inconsistent waddling gait]; 2 = moderate change [moderate ataxia with consistent waddling gait]; 3 = severe change [severe ataxia with reduced righting response]; 4 = spastic paresis of hind legs). The final score (0 to 10 points) was calculated by summarizing the scores of the aforementioned parameters. Animals reaching the maximum of points in one or multiple parameters were excluded from the experiment and euthanized immediately for animal welfare purposes.

RotaRod^®^ performance tests for the quantification of motor coordination was conducted on an accelerating rod (TSE Systems GmbH, Bad Homburg v. d. Höhe, Germany) from five rounds per minute (rpm) to 55 rpm over a maximum period of five minutes. The rpm value was automatically recorded when the mice fell off the rod. Three measurements per animal were performed and obtained values averaged [48].

### Necropsy and sampling

At the defined time points illustrated in Fig 1, animals were euthanized with medetomidine (1.0 mg/kg) and ketamine (200 mg/kg) i.p. under general anesthesia. Samples for complete histological examination (organs see below), immunohistochemistry (spinal cord, intestine), *in situ* hybridization (ISH, spinal cord, intestine), flow cytometry (spleen), and reverse transcription-quantitative polymerase chain reaction (RT-qPCR, spinal cord, spleen) were taken at 7, 14, 22, and 42 dpi (experiment I) and 42 and 49 dpi (experiment II), respectively. From experiment III (non-infectious conditions) samples for histology (organs see below), immunohistochemistry (intestine) and flow cytometry (spleen) were taken at day 7, 14, and 21.

At necropsy, spleen tissue from each animal was processed for flow cytometry. In addition, parts of spleen and spinal cord tissue (cervical, thoracic and lumbar segments) were immediately snap frozen for molecular analyses. Remaining spinal cord segments and spleen tissue as well as thymus, mesenteric lymph node (LN), liver, stomach, pancreas, trachea, esophagus, thyroid gland, heart, lung, kidney, adrenal gland, ovaries, uterus, urinary bladder, tongue, salivary gland, haired skin, eyes, skeletal muscle, femur and sternum, small intestine (two cross sections), cecum (one cross section) and colon (five cross sections) were fixed in formalin for 24 h and embedded in paraffin wax for histology. Bony tissue (femur, sternum) was decalcified for 48h with 10 % disodium-ethylenediaminetetraacetate prior to embedding. Paraffin embedded tissues of spinal cord and intestine were also used for immunohistochemistry and *in situ* hybridization. Serial sections (2–4 μm) were cut and affixed to coated glass slides (Superfrost Plus® slides, Menzel Co., Braunschweig, Germany).

### Histology

Sections of all organs were stained with hematoxylin and eosin (H&E) and were examined by routine light microscopy for the presence of inflammatory changes by two examiners (AU, AB), blinded to the treatment group. In addition, inflammatory responses in the small intestine, cecum and colon were scored by using a four-part, semiquantitative scoring system, modified from Tamaki et al. [51] including the following parameters: severity (0 = none, 1 = mild, 2 = moderate and 3 = severe infiltration) and extent of inflammation (0 = none, 1 = inflammation restricted to mucosa, 2 = inflammation of mucosa and submucosa, 3 = transmural inflammation affecting all layers), percentage of area affected (0 = 0 %, 1 = 1–25 %, 2 = 26–50 %, 3 = 51–75 % and 4 = 76–100 %) and extent of crypt damage (0 = none, 1 = ≤ basal ⅓ affected, 2 = basal ⅔ affected, 3 = crypts lost, surface epithelium present, 4 = crypts and surface epithelium lost). The scores of the four parameters were added up for each cross section (score: 0–14) and a final score was calculated by averaging the segments.

Leukomyelitis was graded in cervical, thoracic and lumbar spinal cord cross sections using a two-part, semiquantitative scoring system (leukomyelitis score) including perivascular infiltrates (PVI, 0 = no changes; 1 = scattered PVI, 2 = two to three layers, 3 = more than three layers of PVI) and hypercellularity (1 = 1–25 cells, 2 = 26–50 cells, 3 = > 50 cells per high power field) as previously described [44, 47, 48, 52]. Arithmetic averages were calculated for each segment and each parameter and a final score was determined by averaging the scores of the three segments from each animal and adding the scores of PVI and hypercellularity.

### Immunohistochemistry

Immunohistochemistry (IHC) was performed using a polyclonal rabbit anti-CD3-specific Ab (DakoCytomation, Hamburg, Germany) for detecting T cells, a rat anti-CD45R/B220-specific monoclonal Ab (BD Biosciences, Heidelberg, Germany) for detecting B cells, a polyclonal rabbit anti-TMEV-specific Ab for detecting TMEV-Ag and a rat anti-*forkhead box P3* (Foxp3)-specific monoclonal Ab (eBioscience, Frankfurt, Germany) for detecting Treg [36, 44, 48, 52–54]. Additionally, a rat anti-CD107b-specific monoclonal Ab (AbD Serotec, Oxford, UK) and a polyclonal goat anti-arginase-1-specific Ab (Santa Cruz Biotechnology Inc., Heidelberg, Germany) for detecting macrophages/microglia and M2-type (modulatory) macrophages/microglia, respectively, were used on spinal cord tissue [36, 48, 55, 56]. Damaged axons were labeled with a goat anti-non-phosphorylated neurofilament (np-NF)-specific monoclonal Ab (Biolegend, London, UK) and the amount of myelin basic protein (MBP) was determined by using a polyclonal rabbit anti-MBP-specific Ab (Chemicon International, Hofheim am Taunus, Germany) [44, 48, 52, 57, 58]. In the colon, phenotypical changes were determined using Foxp3-, CD3-, and CD45R/B220-specific antibodies. Macrophages in the colonic mucosa were quantified by the use of an ionized calcium binding adaptor molecule 1 (Iba-1)-specific polyclonal Ab (Wako Pure Chemical Industries, Richmond, USA). All reactions were conducted as previously described and summarized in Table 1.

**Table 1:** Summary of antibodies used for immunohistochemistry.

Ab	Primary Ab	Pre-treatment and dilution	Specifity
**CD3** A0452	DakoCytomation polyclonal Ab rabbit	Citrate buffer / microwave 1:1000	T cells
**CD45R/B220** 553085 clone RA3-6B2	BD Biosciences monoclonal Ab rat	Citrate buffer / microwave 1:1000	B cells
**Foxp3** 14-5773 clone FJK-16s	eBioscience monoclonal Ab rat	Citrate buffer / microwave 1:50	Treg
**CD107b** MCA2293B clone M3/84	AbD Serotec monoclonal Ab rat	Citrate buffer / microwave 1:200	Activated macrophages/microglia
**Iba-1** 019-19741	Wako Pure Chemical Industries polyclonal Ab rabbit	Citrate buffer / microwave 1:500	Macrophages
**Arginase-1** sc-18351	Santa Cruz Biotechnology polyclonal Ab goat	Citrate buffer / microwave 1:50	Activated M2-type macrophages / microglia
**Np-NF** 801703 clone SMI 311	BioLegend monoclonal Ab goat	Citrate buffer / microwave 1:8000	Axonal damage
**MBP** AB980	Merck Millipore polyclonal Ab rabbit	No pretreatment 1:2000	MBP
**TMEV**	Kummerfeld et al. 2009 polyclonal Ab rabbit	No pretreatment 1:2000	TMEV BeAn

Ab = antibody, Foxp3 = forkhead box p3; Treg = regulatory T cells; Iba-1 = ionized calcium binding adaptor molecule 1; np-NF = non phosphorylated neurofilament; MBP = myelin basic protein; TMEV = Theiler’s murine encephalomyelitis virus.

For evaluating CD3, CD45R/B220, Foxp3, TMEV, CD107b and arginase-1 the absolute number of immunoreactive cells was counted in all three spinal cord segments of each animal. The MBP-negative white matter area was measured by manual mapping at 100x magnification using the fluorescence microscope BZ-9000E (Keyence, Mechelen, Belgium), the BZ-II analyzer software (BZ-H2AE, Keyence, Mechelen, Belgium), and the area measurement function. For determining np-NF-positive axons the total numbers in the white matter of all three spinal cord segments were enumerated.

In the colon, Foxp3^+^ (Treg), CD3^+^ (T cells), CD45R/B220^+^ (B cells), and iba-1^+^ cells (macrophages) infiltrating the *lamina propria* were counted in ten randomly chosen high power fields (magnification 400x) and numbers averaged. Additionally, TMEV-specific IHC was performed on all sections of intestine from infected animals to rule out enteric infection.

### In situ hybridization

ISH for detection of TMEV-specific RNA on cross sections of intestine was performed as described previously [45, 59]. Spinal cord tissues of TMEV-infected and non-infected animals were used as positive and negative controls, respectively. Summarized, a PCR product was generated from a TMEV-infected baby hamster kidney cell culture by RT-qPCR and use of the TMEV-specific primer pair shown in Table 2. Afterwards the PCR product was cloned into a PCR 4-TOPO plasmid vector and amplified in DH5α-T1^®^ cells (TOPO TA Cloning Kit for sequencing, Invitrogen, Karlsruhe, Germany). The *in vitro* transcription was accomplished with DIG-RNA-labeling Mix and T3- and T7-RNA-polymerases (Roche Diagnostics, Mannheim, Germany) according to the manufacturer’s protocol. Formalin-fixed paraffin-embedded tissue sections were dewaxed and washed in diethylpyrocarbonate (DEPC)-treated water (0.1%, Sigma-Aldrich Chemie, Taufkirchen, Germany) and endogenous peroxidase activity was blocked with levamisol. After proteolysis with Proteinase K (1 μg/ml, Roche Diagnostics, Mannheim, Germany), acetylation and prehybridization, hybridization was realized overnight with a probe concentration of 200 ng/ml. The detection system was composed of an anti-DIG-oxigenin-antibody conjugated with alkaline phosphatase (1:200, Roche Diagnostics, Mannheim, Germany), 5-bromo-4-chloro-3-indolyl phosphate (Sigma-Aldrich Chemie, Taufkirchen, Germany) and nitroblue tetrazolium chloride (Sigma-Aldrich Chemie, Taufkirchen, Germany).

**Table 2:** Summary of primer pairs used for polymerase chain reaction.

Gene	primer	sequence 5‘ → 3‘	length
**IL-1α**	forward	AAG CAA CGG GAA GAT TCT GA	179 bp
	reverse	TGA CAA ACT TCT GCC TGA CG	
**IL-2**	forward	GCA GGA TGG AGA ATT ACA GGA	183 bp
	reverse	TGA AAT TCT CAG CAT CTT CCA A	
**IL-4**	forward	CCT CAC AGC AAC GAA GAA CAC C	133 bp
	reverse	CAT CGA AAA GCC CGA AAG AGT C	
**IL-5**	forward	ATG GAG ATT CCC ATG AGC AC	180 bp
	reverse	CCC ACG GAC AGT TTG ATT CT	
**IL-6**	forward	GTT CTC TGG GAA ATC GTG GA	176 bp
	reverse	CCA GAG GAA ATT TTC AAT AGG C	
**IL-10**	forward	CCA AGC CTT ATC GGA AAT GA	162 bp
	reverse	TTT TCA CAG GGG AGA AAT CG	
**Foxp3**	forward	TTC-TCA-CAA-CCA-GGC-CAC-TTG	88 bp
	reverse	CCC-AGG-AAA-GAC-AGC-AAC-CTT	
**TNF**	forward	GCC TCT TCT CAT TCC TGC TT	203 bp
	reverse	CAC TTG GTG GTT TGC TAC GA	
**IFNγ**	forward	CAC GGC ACA GTC ATT GAA AG	144 bp
	reverse	AAT CTG GCT CTG CAG GAT TT	
**TGF-β1**	forward	TTG CTT CAG CTC CAC AGA GA	183 bp
	reverse	TGG TTG TAG AGG GCA AGG AC	
**TMEV**	forward	GAC TAA TCA GAG GAA CGT CAG C	129 bp
	reverse	GTG AAG AGC GGC AAG TGA GA	
**MBP**	forward	GAC TCA CAC ACG AGA ACT AC	117 bp
	reverse	GTG TTC GAG GTG TCA CAA	
**GAPDH**	forward	GAG GCC GGT GCT GAG TAT GT	288 bp
	reverse	GGT GGC AGT GAT GGC ATG GA	
**HPRT**	forward	GGA CCT CTC GAA GTG TTG GA	169 bp
	reverse	TTG CGC TCA TCT TAG GCT TT	
**β-actin**	forward	GGC TAC AGC TTC ACC ACC AC	233 bp
	reverse	ATG CCA CAG GAT TCC ATA CC	

IL = interleukin; bp = base pairs; Foxp3 = forkhead box p3; TNF = tumor necrosis factor; IFN = interferon; TGF = transforming growth factor; TMEV = Theiler’s murine encephalomyelitis virus; MBP = myelin basic protein; GAPDH = glyceraldehyde-3-phosphate dehydrogenase; HPRT = Hypoxanthine-guanine phosphoribosyltransferase.

### RNA isolation and reverse transcription

RNA was isolated from 10 to 40 mg of snap frozen spinal cord and spleen from TMEV- and mock-infected animals using an Omni´s PCR Tissue Homogenizing Kit (Süd-Laborbedarf GmbH, Gauting, Germany), QIAzol^TM^ Lysis Reagent (Qiagen GmbH, Hilden, Germany) and a RNeasy^®^ Mini Kit (Qiagen GmbH, Hilden, Germany) according to the manufacturer´s protocols. Subsequently, equal amounts of RNA were transcribed into cDNA with the Omniscript^TM^ RT Kit (Qiagen GmbH, Hilden, Germany), RNAseOut^TM^ Recombinant Ribonuclease Inhibitor (Invitrogen^TM^ GmbH, Karlsruhe, Germany) and Random Primers (Promega GmbH, Mannheim, Germany) as described previously [47, 60].

### Reverse transcription-quantitative polymerase chain reaction

RT-qPCR was performed for TMEV, IL-1α, IL-2, IL-4, IL-5, IL-6, IL-10, Foxp3, tumor necrosis factor (TNF), interferon (IFN)-γ, transforming growth factor (TGF)-β1, MBP and three reference genes (glyceraldehyde 3-phosphate dehydrogenase, β-actin, hypoxanthin-guanin-phosphoribosyltransferase) in spinal cord and spleen tissue by use of the Mx3005P Multiplex Quantitative PCR System and Brilliant III Ultra-Fast SYBR^®^ Green RT-qPCR Master Mix (Agilent Technologies Deutschland, Böblingen, Germany). Primer sequences for IL-5 and IL-6 were determined using the Primer3 input software. All other used sequences were taken from the literature [36, 47, 48, 61, 62]. Primer sequences are listed in Table 2. For quantification ten-fold serial dilution standards ranging from 10^8^ to 10^2^ copies/μl were prepared. The normalization factor for correction of experimental variations was calculated from the three reference genes with geNorm software version 3.4 [63].

### Flow cytometry

For phenotypical analysis of peripheral leukocytes, spleen samples were dissolved to single cell suspension in phosphate-buffered saline containing 0.2 % BSA (Gibco, Darmstadt, Germany). Erythrocytes were lysed by adding Ammonium-Chloride-Potassium Lysing Buffer (Gibco, Darmstadt, Germany) and the cell number was determined using trypan blue solution (Sigma Aldrich Chemie GmbH, Taufkirchen, Germany) and a Neubauer Improved chamber. To avoid unspecific binding to Fc receptor, preincubation with anti-CD16/CD32 Ab (clone 2.4G2; BioXCell, West Libanon, USA) was performed, and dead cells were stained using the LIVE/DEAD® fixable dead cell stain kit (Invitrogen^TM^ GmbH, Karlsruhe, Germany) as described by the manufacturer. Subsequently, anti-CD3ε (PE-Cy7; clone 145-2C11; eBioscience, San Diego, USA), anti-CD4 (PacificBlue; clone GK1.5; BioLegend, London, UK), anti-CD8α (HV500; clone 53–6.7; BD Biosciences, Heidelberg, Germany), anti-CD19 (PE-eFluor610; clone 1D3; eBioscience, San Diego, USA), anti-CD69 (FITC; clone H1.2F3; BioLegend, London, UK) and anti-CD44 Ab (APC; clone IM7; BioLegend, London, UK) were added for fluorochrome-conjugated surface marker staining. For intracellular staining of Foxp3 the Foxp3 Transcription Factor Staining Buffer Set (eBioscience, San Diego, USA) was used according to the manufacturer´s protocol. Samples were acquired with an LSRII SORP cytometer (BD Biosciences, Heidelberg, Germany) and analyzed using FlowJo software version 9.6.4 (Tree Star, Ashland, USA). For gating strategy see S1–S3 Figs.

### Statistical analysis

All statistical analyses were conducted using Statistical analysis software SAS 9.3 and the Enterprise Guide 5.1 for Windows (SAS Institute Inc., Cary, NC, USA). Data generated from several mice from each group were analyzed using Kruskal-Wallis tests. Relation of two groups was performed by use of multiple Wilcoxon rank-sum tests. Results were considered statistically significant at p-value < 0.05. The geometric mean of fluorescence intensity (gMFI) for CD69 and CD44 in CD4^+^ and CD8^+^ T cells was determined using the FlowJo software version 9.6.4 (Tree Star, Ashland, USA). Box and whisker plots were generated with GraphPad Prism 6.0 (GraphPad Software, La Jolla, USA) and display median, minimum and maximum values as well as upper and lower quartiles.

## Results

### Experiments I and II: interleukin-10 receptor blockade in acute and chronic Theiler’s murine encephalomyelitis

#### Interleukin-10 receptor blockade causes clinical deterioration without influencing motor coordination in Theiler’s murine encephalomyelitis virus infected SJL mice

Clinical examination of mice, including RotaRod® performance test and scoring of clinical parameters was performed to characterize neurological and systemic signs caused by TMEV-infection and IL-10R blockade, respectively.

**Fig 2:**
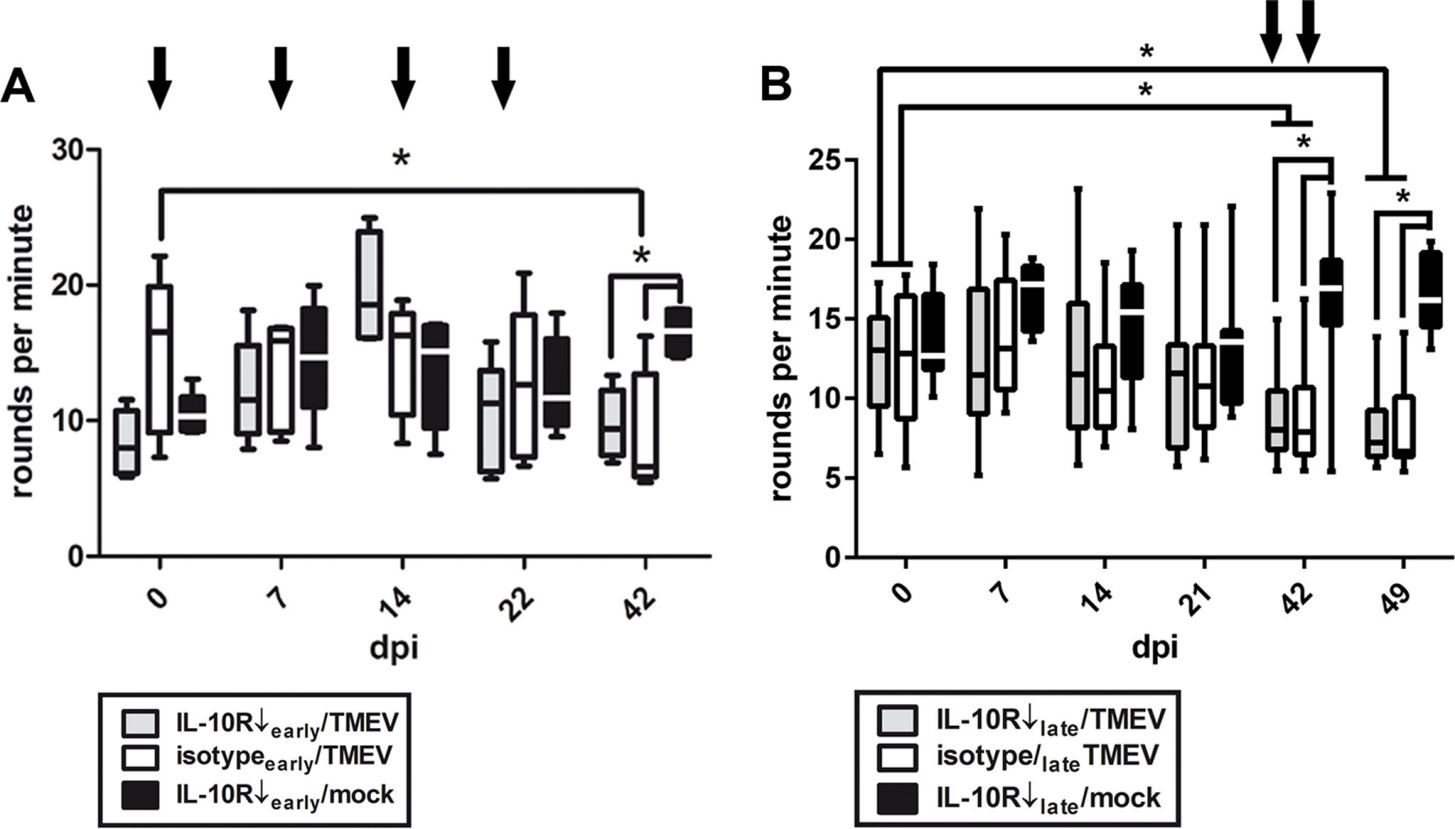
Motor coordination in Theiler’s murine encephalomyelitis virus (TMEV)-infected SJL mice. (A) *Experiment I*: RotaRod^®^ performance test after early application of the IL-10 receptor blocking antibody (IL-10R Ab) at 0, 7, 14 and 21 dpi (arrows) revealed motor coordination deficits in TMEV-infected animals compared to mock-infected mice at 42 dpi. However, no differences were determined between IL-10R Ab- and isotype-treated animals. white box = TMEV-infected mice without IL-10R Ab treatment (group “isotype_early_/TMEV”), grey box = TMEV-infected mice with IL-10R treatment (group “IL10R↓_early_/TMEV”), black box = mock-infected mice with IL-10R treatment (group “IL10R↓_early_/mock”). (B) *Experiment II*: application of IL-10R Ab at 35 and 42 dpi (arrows) caused also deterioration of motor coordination starting at 42 dpi. However, no differences were observed between IL-10R Ab- and isotype-treated animals (groups “IL-10R↓_late_/TMEV” and “isotype_late_/TMEV”). white box = TMEV-infected mice without IL-10R Ab treatment (group “isotype_late_/TMEV”), grey box = TMEV-infected mice with IL-10R treatment (group “IL10R↓_late_/TMEV”), black box = mock-infected mice with IL-10R treatment (group “IL10R↓_late_/mock”). Box and whisker plots display median, minimum and maximum values as well as upper and lower quartiles, 5 animals used at all investigated time points, Wilcoxon rank-sum tests, *  =  p < 0.05.

In experiment I clinical examination of acutely infected mice was performed at 0, 7, 14, 22, and 42 dpi. Intracerebral TMEV-infection induced ataxia and motor coordination deficits determined by RotaRod^®^ performance test at 42 dpi in mice with and without IL-10R blockade (Fig 2). However, no differences were detected between these two groups (groups “IL-10R↓_early_/TMEV” and group “isotype_early_/TMEV”) at 42 dpi (Fig 2, Table A in S1 File), suggestive of minor or absent effects of IL-10R blockade upon motor coordination and spinal cord pathology, respectively. As expected [48], no motor coordination deficits were observed in non-infected mice following IL-10R blockade (group “IL10R↓/mock”) at any investigated time point (Fig 2, Table B in S1 File). No fatalities were observed in any treatment group.

**Fig 3:**
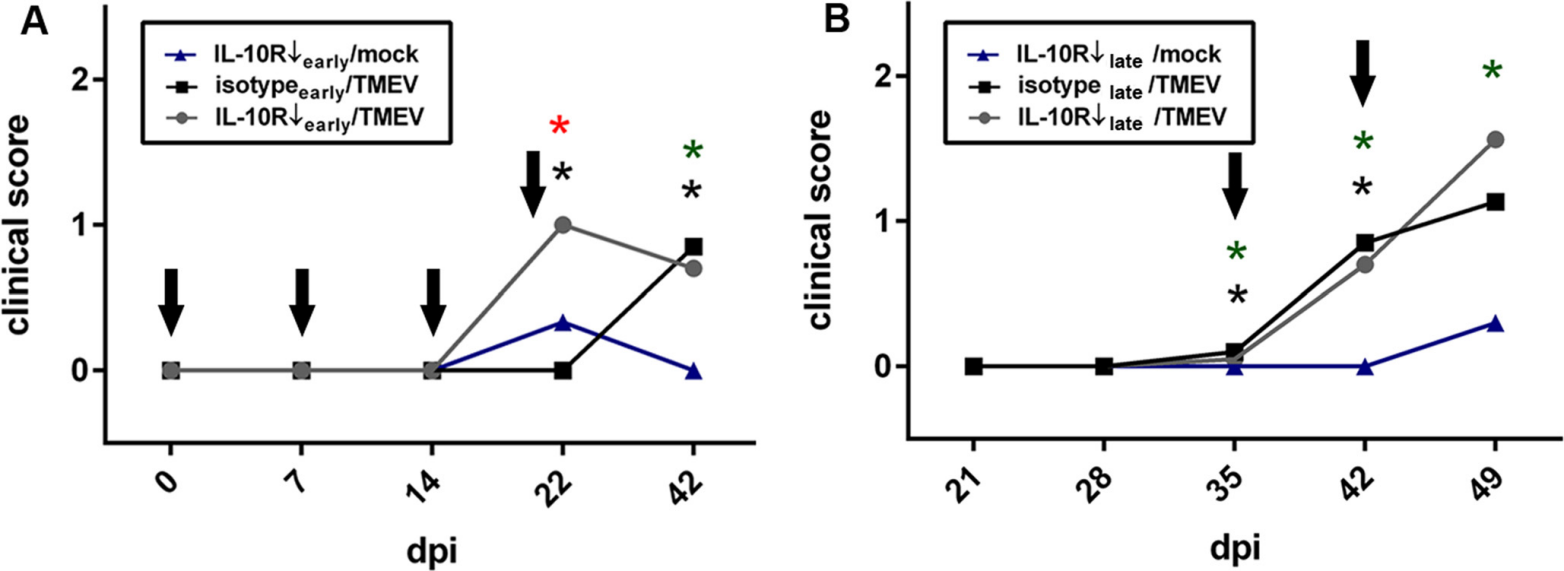
Clinical effects of Theiler’s murine encephalomyelitis virus (TMEV)-infection in SJL mice following IL-10R blockade. (A) *Experiment I*: TMEV-infection causes worsening of systemic clinical signs (increased clinical scores) at 22 dpi in SJL mice with early anti-IL-10 receptor antibody (IL-10R Ab) treatment compared to non-infected mice following anti-IL-10R Ab treatment (**red asterisks**) and TMEV-infected mice without anti-IL-10R Ab treatment (**black asterisks**), suggestive of a triggering effect of virus infection upon IL-10R deficiency-mediated systemic signs. (B) *Experiment II*: Ab treatment during the late infection phase leads to similar clinical scores between TMEV-infected mice with and without IL-10R blockade. 5 animals used in all three groups and at all investigated time points, Wilcoxon rank-sum tests, arrows = administration of IL-10R Ab or isotype control, respectively, red asterisks = significant differences (p < 0.05) between TMEV-infected mice with and without IL-10R blockade, black asterisks = significant differences (p < 0.05) between IL-10R blocked mice with and without TMEV-infection, green asterisks significant differences (p < 0.05) between TMEV-infected mice and IL-10R blocked mice.

Clinical scoring revealed that IL-10R Ab treatment (groups “IL-10R↓_early_/TMEV” and “IL-10R↓_early_/mock”) during the early infection phase caused increased clinical scores at 22 dpi compared to animals, that received isotype control Ab only (group “isotype_early_/TMEV”; Fig 3, Table B in S1 File). Affected animals displayed reduced activity, hunched backs and scurfy hair, suggestive of systemic clinical signs. Noteworthy, unformed feces ascribed to enteric disease were observed in Ab-treated mice. Strikingly, the clinical score at 22 dpi was significantly increased in TMEV-infected mice (group “IL-10R↓_early_/TMEV”) compared to mock-infected animals (group “IL-10R↓_early_/mock”) following anti-IL-10R Ab treatment, suggestive of a triggering or additive effect of acute virus infection upon IL-10R deficiency-mediated systemic signs (Fig 3, Table B in S1 File). At 42 dpi, increased clinical scores were observed in IL-10R-blocked infected mice (group “IL-10R↓_early_/TMEV”) and infected mice that received isotype control Ab (group “isotype_early_/TMEV”), showing that systemic clinical signs are primarily a consequence of virus infection at later time points. Obtained data of experiment I are summarized in Tables C-E in S1 File.

Experiment II was executed to determine whether the triggering effect of virus infection upon IL-10R deficiency-mediated signs depends upon the disease phase. RotaRod^®^ performance test was performed at 0, 7, 14, 21, 42, and 49 dpi in animals with Ab treatment during the later chronic TME phase. A significant decline of motor coordination was found at 42 and 49 dpi in mice with and without IL-10R blockade (Fig 2, Table F in S1 File; groups “10R↓_late_/TMEV” and “isotype_late_/TMEV”). However, no differences were detected between group “IL-10R↓_late_/TMEV” and group “isotype_late_/TMEV” mice (Fig 2, Table F in S1 File). Similar to experiment I, no neurological defects were observed in non-infected mice following Ab treatment (group “10R↓_late_/mock”; Table G in S1 File).

Clinical soring revealed significantly increased scores in infected mice at 42 and 49 dpi after late Ab application. However, no differences were determined between both groups (groups “10R↓_late_/TMEV” and “isotype_late_/TMEV”) at these time points (Fig 3, Table F in S1 File), showing that chronic virus infection has only a limited ability to trigger IL-10R deficiency-mediated effects. Obtained data of experiment II are summarized in Tables C-E in S1 File.

In summary, results show that IL-10R neutralization causes systemic clinical signs in SJL mice which are temporarily (22 dpi) enhanced by acute TMEV-infection (experiment I). Conversely, IL-10R blockade is insufficient to alter neurological deficits (motor coordination) associated with TMEV-induced demyelinating disease, as no differences in motor coordination were observed between infected mice with and without Ab treatment (experiment I and II).

#### Interleukin-10 receptor blockade leads to an enhanced recruitment of T cells to the spinal cord during the demyelinating phase of Theiler’s murine encephalomyelitis but does not influence the virus load

Clinical examinations revealed only negligible effects of IL-10R blockade upon the CNS of TMEV-infected mice. In order to determine more subtle changes, spinal cord tissues were evaluated by histology, IHC and RT-qPCR.

**Fig 4:**
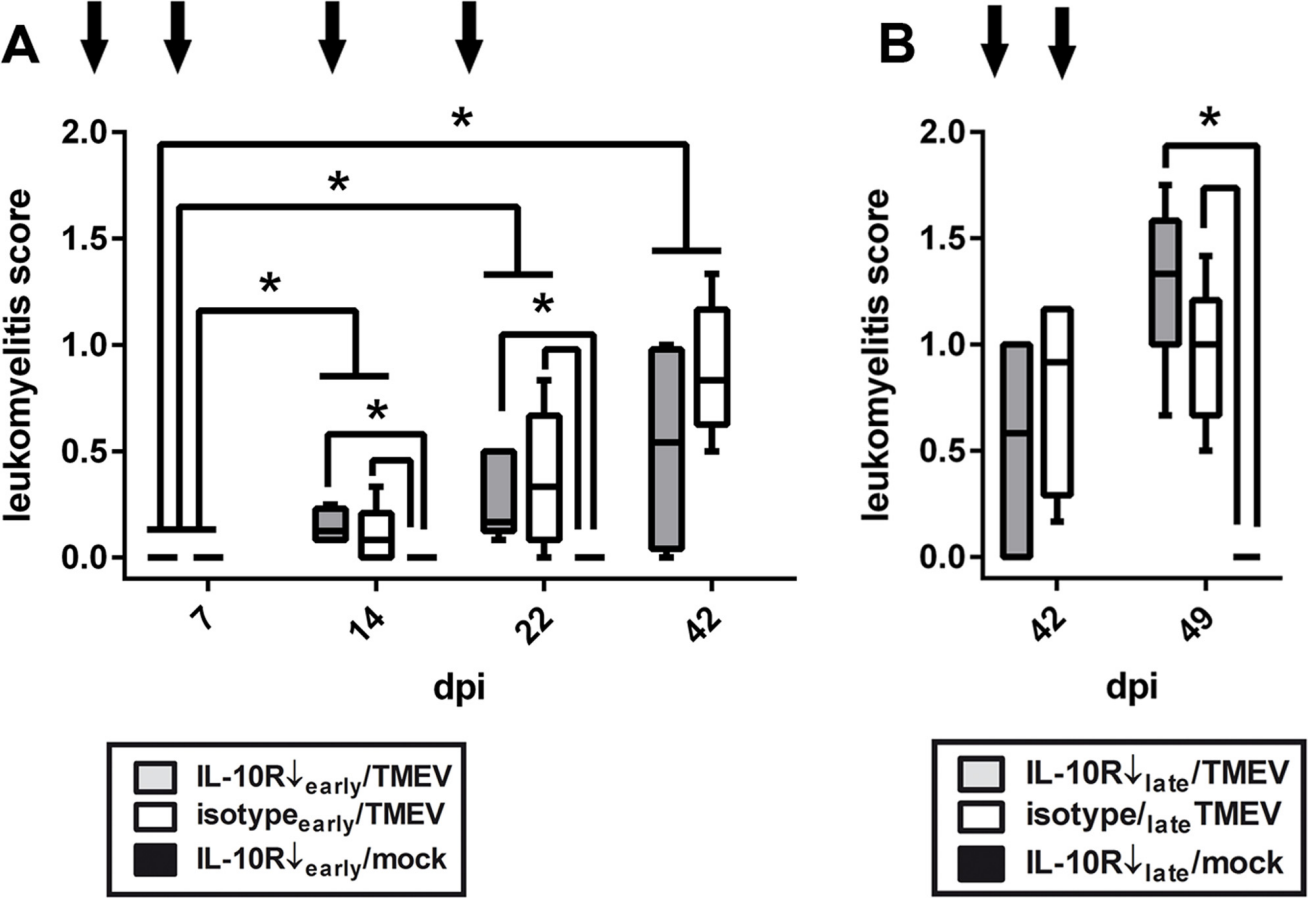
Leukomyelitis in Theiler’s murine encephalomyelitis virus (TMEV)-infected SJL mice. (A) *Experiment I*: histological scoring of the spinal cord revealed progressive leukomyelitis in all TMEV-infected animals starting at 14 dpi. No inflammation occurred in mock-infected animals and no differences were observed between TMEV-infected animals receiving anti-IL-10 receptor antibody (IL-10R Ab) or isotype control. Arrows indicate antibody treatment at 0, 7, 14 and 21 dpi in the early phase of TME. white box = TMEV-infected mice without IL-10R Ab treatment (group “isotype_early_/TMEV”), grey box = TMEV-infected mice with IL-10R treatment (group “IL10R↓_early_/TMEV”), black box = mock-infected mice with IL-10R treatment (group “IL10R↓_early_/mock”). (B) *Experiment II*: chronic phase application of IL-10R Ab at 35 and 42 dpi (arrows) did not cause differences between TMEV-infected animals receiving IL-10R Ab or isotype control. white box = TMEV-infected mice without IL-10R Ab treatment (group “isotype_late_/TMEV”), grey box = TMEV-infected mice with IL-10R treatment (group “IL10R↓_late_/TMEV”), black box = mock-infected mice with IL-10R treatment (group “IL10R↓_late_/mock”). Box and whisker plots display median, minimum and maximum values as well as upper and lower quartiles, 5 animals used at all investigated time points, Wilcoxon rank-sum tests, *  =  p < 0.05.

Our previous studies in SJL mice have demonstrated the occurrence of progressive leukomyelitis with TMEV-induced demyelination starting at 28 dpi [44, 45]. To characterize the impact of IL-10R blockade during the acute, initial TME phase, Ab were administered before the onset of demyelination and spinal cord tissue were evaluated by histology and IHC at 7, 14, 22, and 42 dpi (experiment I). Histological scoring revealed significant inflammatory responses at 14, 22, and 42 dpi in mice with and without Ab treatment (Fig 4, Table A in S1 File; groups “IL-10R↓_early_/TMEV” and “isotype_early_/TMEV”). However, no differences were observed between these two groups at any time point (Fig 4). Non-infected controls with IL-10R blockade (group “10R↓_early_/mock”) included at 14 and 22 dpi showed no spinal cord inflammation (Fig 4, Table B in S1 File).

**Fig 5:**
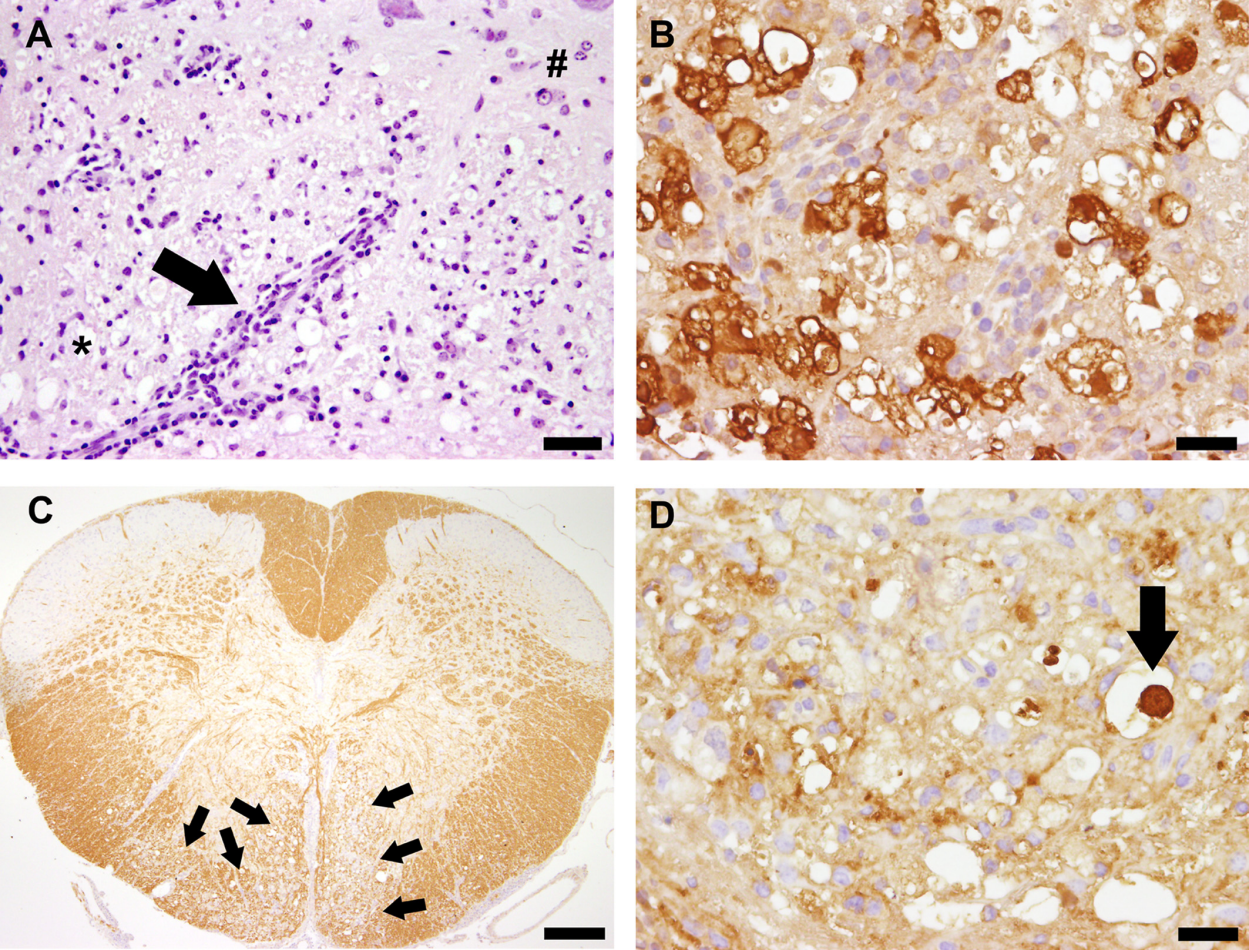
Neuropathological findings in Theiler’s murine encephalomyelitis virus (TMEV)-infected SJL mice. (A) Leukomyelitis in a TMEV-infected animal at 49 days post infection (dpi). Note perivascular and meningeal (arrow) infiltration of inflammatory cells within the white (*) and grey matter (#). H&E staining, bar = 60 μm. (B) Arginase-1^+^ M2-type microglia/macrophages with gitter cell morphology at 49 dpi, indicative of myelinophagia. Immunohistochemistry, bar = 20 μm. (C) Bilateral loss (arrows) of myelin basic protein (demyelination) in ventral funiculi of a cervical spinal cord segment at 49 dpi., Immunohistochemistry, bar = 200 μm. (D) Axonal damage characterized by axonal swelling (spheroid formation) with accumulation of np-NF (arrow) in the spinal cord white matter at 49 dpi. Immunohistochemistry, bar = 20 μm.

Spinal cord inflammation was characterized by an infiltration of CD3^+^ T cells, CD45R/B220^+^ B cells, Foxp3^+^ Treg, CD107b^+^ microglia/macrophages, and arginase-1^+^ M2-type microglia/macrophages (Fig 5), but in accordance with results from histological scoring, no differences were found between group “IL-10R↓_early_/TMEV” and “isotype_early_/TMEV” animals (Table A in S1 File). In addition, the same degree of spinal cord demyelination, determined by densiometric analyses of the MBP-unstained area (Fig 5), and axonal damage, determined by np-NF-specific IHC (Fig 5), was found in both TMEV-infected groups (Table A in S1 File). As expected no, spinal cord lesions were observed by histology and immunohistochemistry in non-infected mice following Ab treatment (group “IL10R↓_early_/mock”; Fig 4, S1 Fig). Obtained data of experiment I are summarized in Tables C-E in S1 File.

RT-qPCR was performed during the initial phase of virus-induced leukomyelitis at 14 and 22 dpi. The lack of group differences was confirmed, showing similar messenger RNA (mRNA) expression levels of Foxp3 and all selected cytokines (IL-1, IL-2, IL-4, IL-5, IL-6, IL-10, TNF, IFN-γ, TGF-β) within the spinal cord of group “IL-10R↓_early_/TMEV” and group “isotype_early_/TMEV” at 14 and 22 dpi (Table A in S1 File). Results show that early systemic IL-10R blockade is unable to significantly influence CNS inflammation in TME.

To determine the impact of IL-10R blockade on antiviral immunity, the amount of TMEV-Ag and -RNA in the spinal cord was measured by IHC and RT-qPCR. Matching the other findings, no differences of virus-protein and -RNA levels were observed between group “IL-10R↓_early_/TMEV” and group “isotype_early_/TMEV” animals (Table A in S1 File). Obtained data of experiment I are summarized in Tables C-E in S1 File.

**Fig 6:**
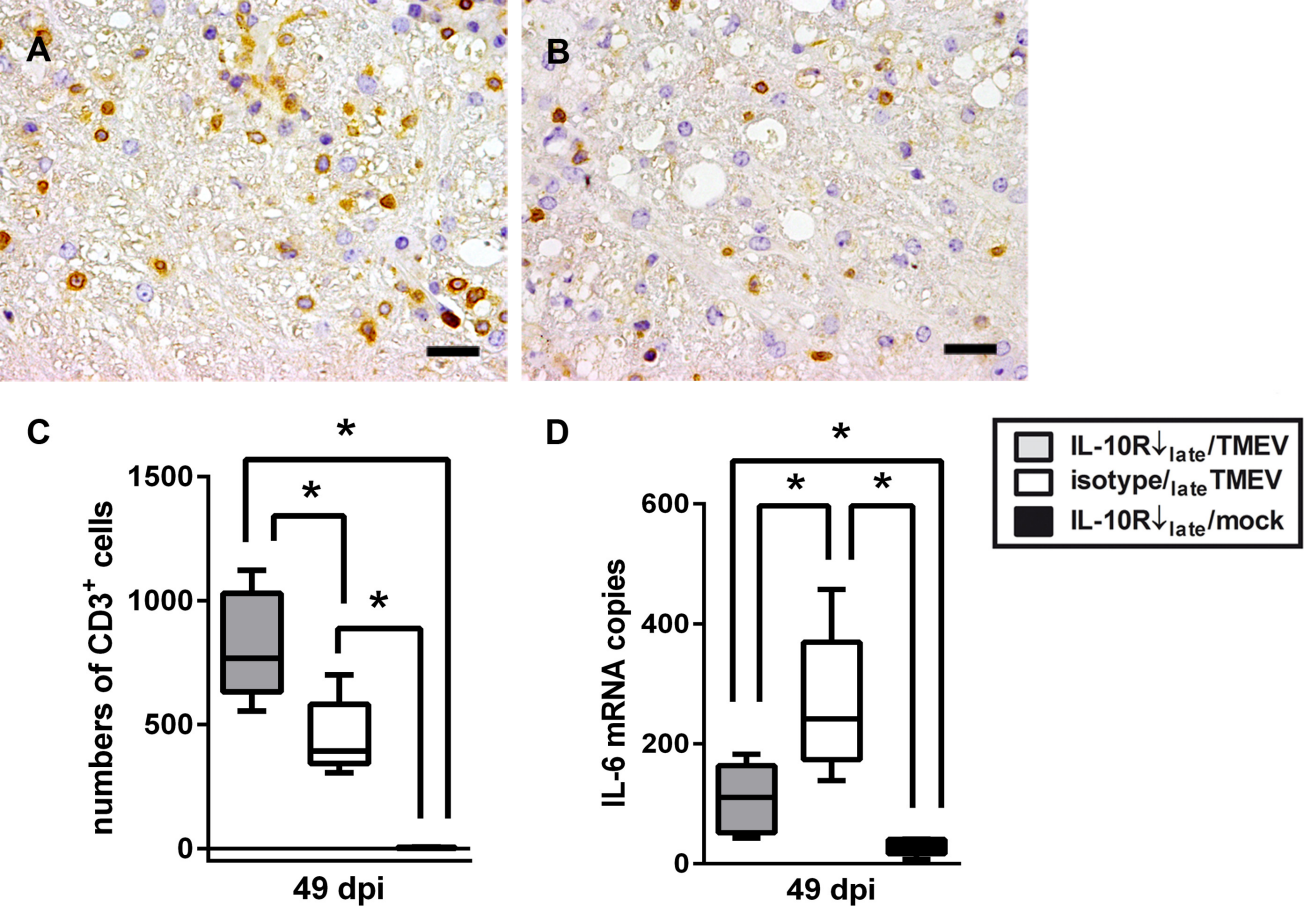
Effect of IL-10 receptor (IL-10R) blockade upon leukomyelitis in Theiler’s murine encephalomyelitis virus (TMEV)-infected SJL mice. (A) TMEV-infection and concurrent intraperitoneal application of IL-10R antibody lead to an increase of CD3^+^ T cells in the spinal cord at 49 days post infection (dpi) compared to (B) TMEV-infected control animals. A,B: immunohistochemistry, bar = 20 μm. (C) Statistical analyses revealed a significant increase of CD3^+^ T cells in TMEV-infected mice with IL-10R blockade compared to isotype-treated animals at 49 dpi. Almost no CD3^+^ T cells were present in spinal cord cross sections of mock-infected animals. (D) Simultaneously, IL-6 mRNA expression significantly decreased in TMEV-infected animals following IL-10R blockade. white box = TMEV-infected mice without IL-10R Ab treatment (group “isotype_late_/TMEV”), grey box = TMEV-infected mice with IL-10R treatment (group “IL10R↓_late_/TMEV”), black box = mock-infected mice with IL-10R treatment (group “IL10R↓_late_/mock”). Box and whisker plots display median, minimum and maximum values as well as upper and lower quartiles, 5 animals used in all groups, Wilcoxon rank-sum tests, *  =  p < 0.05.

Since TME represents a biphasic disease with directly virus-induced tissue damage during the acute stage and immunopathology due to virus persistence during the chronic stage, Ab treatment was performed also at later time points (experiment II). Animals were investigated by histology and IHC in the chronic demyelinating phase at 42 and 49 dpi. Although no differences were observed regarding the severity of leukomyelitis determined by histology (Fig 4), and the severity of demyelination and axonal damage determined by MBP- and np-NF-specific IHC, IHC for CD3 revealed significantly increased numbers of T cells within the spinal cord at 49 dpi (14 days after onset of IL-10R neutralization) in infected mice receiving anti-IL-10R Ab (group “IL-10R↓_late_/TMEV”; Fig 6, Table F in S1 File). Subsequently, spinal cord changes at 49 dpi were further evaluated on a molecular level showing that IL-6 mRNA transcription was significantly decreased in IL-10R blocked mice (group “IL-10R↓_late_/TMEV”) compared to isotype-treated animals following TMEV-infection (group “isotype_late_/TMEV”, Fig 6, Table F in S1 File). However, mRNA expression of IL-1, IL-2, IL-4, IL-5, IL-10, TNF, TGF-β, IFN-γ and Foxp3 remained unchanged between both groups. Moreover, similar to MBP-specific IHC, MBP mRNA-expression showed no group differences (Table F in S1 File). Results indicate a rather mild but not absent effect of IL-10R neutralization upon spinal cord inflammation and pathology. Obtained data of experiment II are summarized in Tables C-E in S1 File.

Similar to experiment I, IL-10R neutralization during the late TME phase did not influence virus concentration in the spinal cord at 42 and 49 dpi, as determined by IHC and RT-qPCR (Table F in S1 File).

Collectively, data indicate that systemic anti-IL-10R blockade is unable to alter antiviral immunity during acute and chronic TME (experiment I and II). However, although being insufficient to influence the severity of neurological signs (motor coordination) and demyelination, increased CD3^+^ T cell influx together with reduced IL-6 transcription in the spinal cord during chronic TME clearly demonstrates a limited but general ability of systemic IL-10R neutralization to alter the inflammatory environment within the CNS (experiment II).

#### Immune-mediated colitis in interleukin-10 receptor blocked mice is aggravated by concurrent Theiler’s murine encephalomyelitis virus-infection

Clinical examination revealed that acute TMEV-infection causes a temporary exacerbation of systemic clinical signs in IL-10R neutralized mice. In order to determine alterations induced by IL-10R deficiency, histological examination of mice with and without IL-10R blockade was performed. Since Ab-mediated pathological changes were largely restricted to the intestine (see experiment III), the degree of colitis was quantified in mice with IL-10R blockade during the acute (experiment I) and chronic (experiment II) TME phase.

In experiment I histology was performed at 7, 14, 22, and 42 dpi in TMEV-infected mice with and without early Ab treatment (comparison group “IL-10R↓_early_/TMEV” vs. “isotype_early_/TMEV”). Colitis was only observed in Ab-treated mice (group “IL-10R↓_early_/TMEV”) and significant differences compared to isotype treated mice (group “isotype_early_/TMEV”) were present at 14, 22, and 42 dpi (Table A in S1 File).

**Fig 7:**
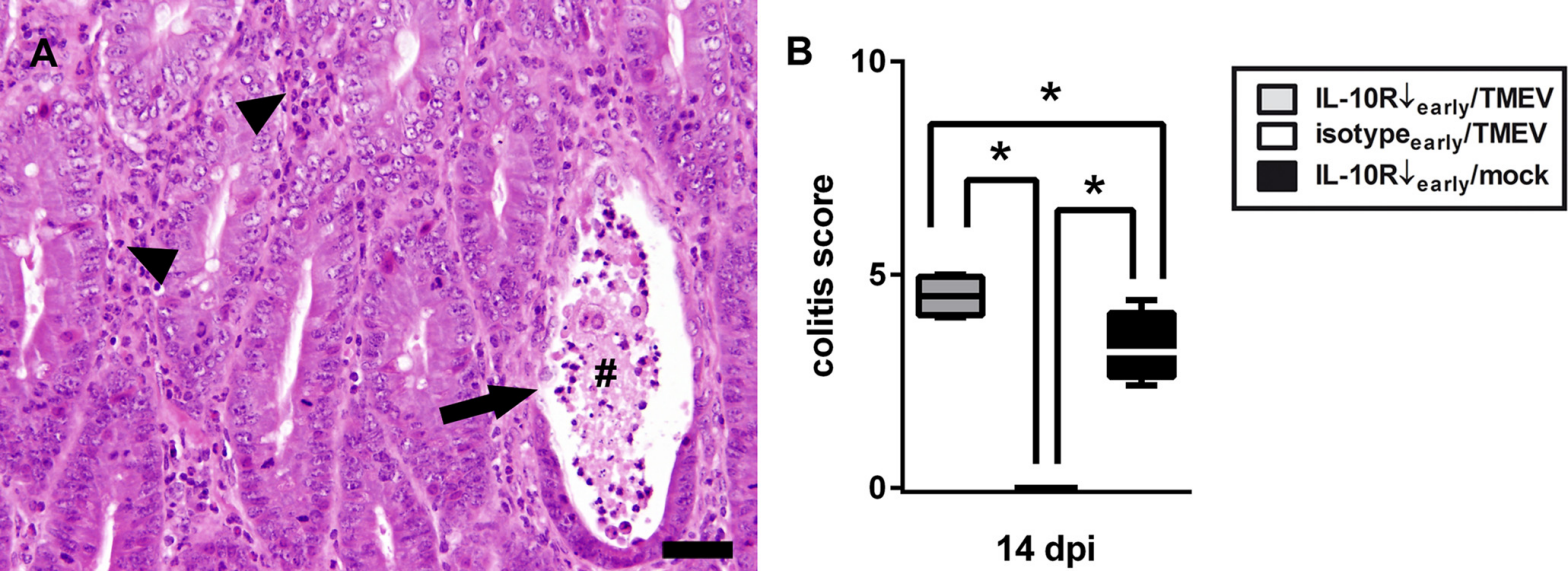
Theiler’s murine encephalomyelitis virus (TMEV)-infection exacerbates enteric disease following interleukin-10 receptor (IL-10R) blockade. (A) Note changes of colonic mucosa with severe lymphohistiocytic to neutrophilic inflammation (arrow heads), crypt abscess formation (#), and loss of crypt epithelium (arrow). H&E staining, bar = 60 μm (B) Significantly increased severity of colitis at 14 days post infection (dpi) in TMEV-infected mice with IL10R antibody (Ab) treatment (group “IL10R↓_early_/TMEV”) compared to Ab treated animals without infection (group “IL10R↓_early_/mock”). Animals receiving isotype control instead of IL-10R Ab did not show any intestinal inflammation. IL-10R Ab treated SJL mice with TMEV-infection (group “IL10R↓_early_/TMEV”), TMEV-infected mice without IL-10R Ab treatment (group “isotype_early_/TMEV”), IL-10R Ab treated SJL mice without TMEV-infection (group “IL10R↓_early_/mock”). Box and whisker plots display median, minimum and maximum values as well as upper and lower quartiles, 5 animals used in all groups, Wilcoxon rank-sum tests, *  =  p < 0.05.

To test the hypothesis that CNS infection exhibits triggering effects upon systemic immunopathology, IL-10R blocked SJL mice were mock- or TMEV-infected and histologically examined at 14 and 22 dpi (comparison group “IL-10R↓_early_/TMEV” vs. group “IL-10R↓_early_/mock”). IL-10R blockade led to colitis at 14 and 22 dpi in infected and non-infected mice. Notably, simultaneous infection of mice treated with Ab during the early TME phase (group “IL-10R↓_early_/TMEV”) resulted in transient worsening of colonic disease compared to mock-infected mice (group “IL-10R↓_early_/mock”) at 14 dpi (Fig 7, Table B in S1 File), characterized by more extensive leukocyte infiltration with hyperplasia of crypt epithelium, increased crypt damage and multifocal transmural inflammation. Obtained data of experiment I are summarized in Tables C-E in S1 File.

TMEV-specific IHC and ISH were performed at 7, 14, 22, and 42 dpi to detect possible direct viral effects on the intestinal tract. Results revealed no viral Ag or RNA in the small intestine, colon, or caecum at any investigated time point in TMEV-infected mice with or without Ab treatment (group “isotype_early_/TMEV” and “IL-10R↓_early_/TMEV”).

In experiment II histology of the colon was performed at 42 and 49 dpi in TMEV-infected mice with and without late Ab treatment. Comparison between group “IL-10R↓_late_/TMEV” and group “isotype_late_/TMEV” revealed significantly increased scores in IL-10R blocked mice at 49 dpi (Table F in S1 File). However, in contrast to experiment I, IL-10R blockade at later time points did not induce group differences at day 14 after Ab treatment onset (49 dpi, Table F in S1 File). As expected, animals that received isotype control Ab (group “isotype_late_/TMEV”) showed no signs of colitis (Table G in S1 File). Obtained data of experiment II are summarized in Tables C-E in S1 File.

Similar to experiment I, no TMEV-Ag or -RNA was detected in the intestinal tract by IHC and ISH at 42 and 49 dpi in TMEV-infected mice with or without Ab treatment (group “isotype_late_/TMEV” and “IL-10R↓_late_/TMEV”; S4 Fig).

Results show that acute TMEV-induced neuroinflammation has the ability to deteriorate immune-mediated colitis in IL-10R neutralized SJL mice (experiment I).

#### Theiler’s murine encephalomyelitis enhances peripheral cytokine expression and phenotypical changes in interleukin-10 receptor blocked SJL mice

To characterize changes of the peripheral immune system associated with IL-10R deficiency-mediated colitis, phenotypical and cytokine expression analyses of spleen tissues were performed during the acute (experiment I) and chronic (experiment II) phase of TME.

In experiment I flow cytometry and RT-qPCR were performed at 7, 14, 22, and 42 dpi in mice with and without IL-10R blockade during acute TME (comparison “IL-10R↓_early_/TMEV” vs. “isotype_early_/TMEV”). Results revealed an increased CD44 expression on CD4^+^ T cells at 14 dpi and an increased frequency of CD19^+^ B cells together with reduced relative numbers of CD4^+^ Foxp3^+^ Treg and an enhanced expression of CD69 on CD4^+^ T cells at 22 dpi in group “IL-10R↓_early_/TMEV” compared to group “isotype_early_/TMEV”). Indicative of increased cellularity, significantly elevated spleen weights were found in IL-10R blocked mice with virus infection compared to isotype treated mice with virus infection at 22 and 42 dpi (Table A in S1 File). Phenotypical changes were associated with an increased transcription of IL-1α (14 and 22 dpi), IL-5 (14 dpi), IL-6 (14 dpi) and TGF-β (14 and 22 dpi), indicative of peripheral immune enhancement following IL-10R neutralization (Table A in S1 File). Obtained data of experiment I are summarized in Tables C-E in S1 File.

**Fig 8:**
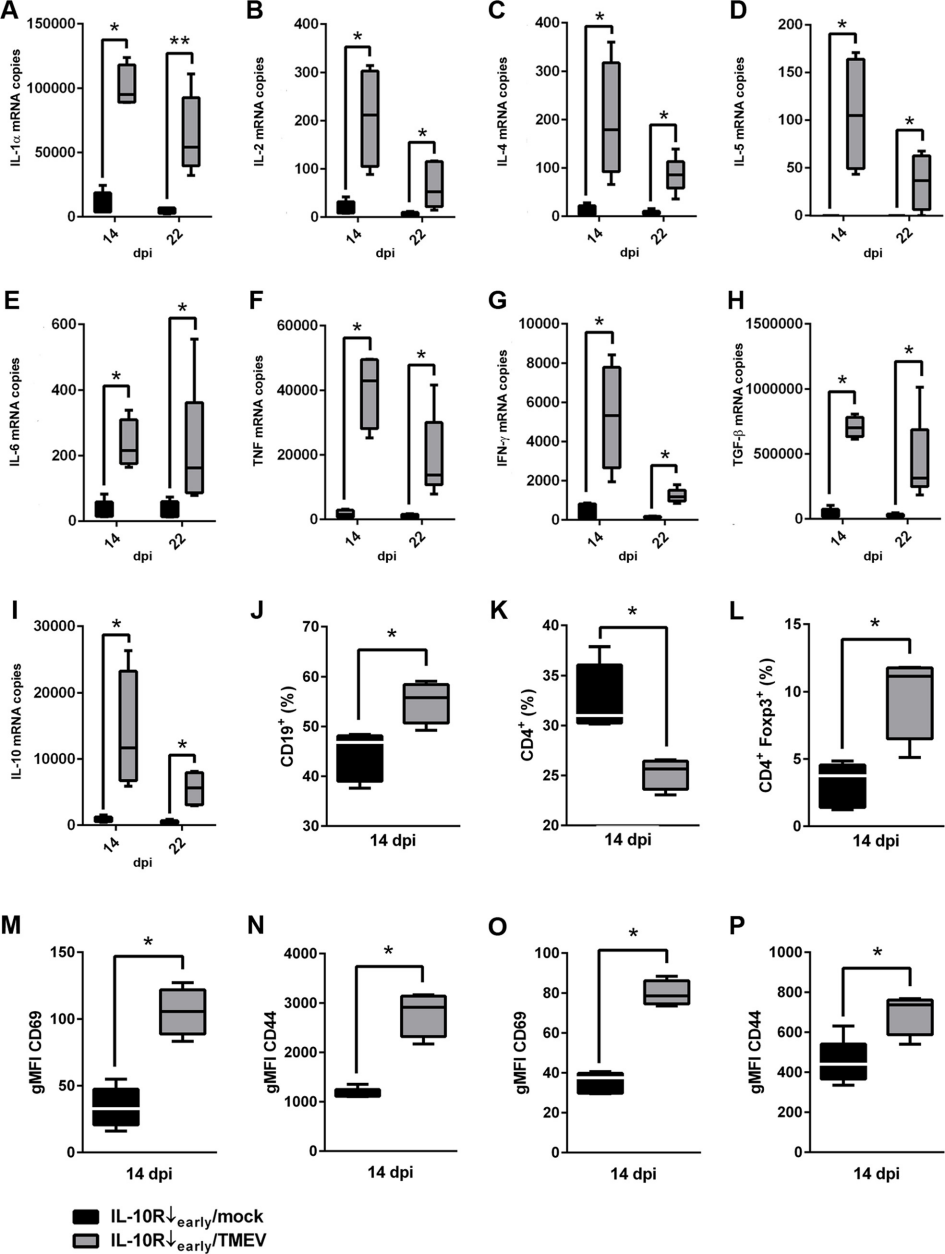
Effects of acute Theiler’s murine encephalomyelitis virus (TMEV)-infection (*experiment I*) upon cytokine expression and phenotypical changes in spleens of interleukin-10 receptor (IL-10R) blocked SJL mice. (A-I) Significantly elevated mRNA levels of IL-1α, IL-2, IL-4, IL-5, IL-6, TNF, IFN-γ, TGF-β and IL-10 in spleens of infected mice (group “IL-10R↓_early_/TMEV”) compared to non-infected animals following IL-10R antibody (Ab) treatment (group “IL10R↓_early_/mock”). (J) Flow cytometry revealed a relative increase of CD19^+^ B cells and a simultaneous decrease of CD4^+^ T cells (K) in the spleen of infected mice following IL-10R blockade at 14 dpi. (L) Additionally, the relative numbers of CD4^+^ Foxp3^+^ Treg in the spleen were increased following TMEV-infection and IL-10R blockade. Moreover, gMFI of CD69 (M) and CD44 (N) gated on CD4^+^ cells and gMFI of CD69 (O) and CD44 (P) gated on CD8^+^ cells were increased at 14 dpi in infected mice following IL-10R Ab treatment. For gating strategy see S1 Fig. IL-10R Ab treated animals without TMEV-infection (group “IL10R↓_early_/mock”). IL-10R Ab treated mice with additional TMEV-infection (group “IL-10R↓_early_/TMEV”). Box and whisker plots display median, minimum and maximum values as well as upper and lower quartiles, 5 animals used in both groups and at all investigated time points, Wilcoxon rank-sum tests, *  =  p < 0.05.

To identify virus-mediated triggering effects upon the peripheral immune system associated with exacerbated immune mediated colitis (14 dpi) and increased clinical scores (22 dpi), phenotypical changes (14 dpi) and cytokine expression (14 and 22 dpi) in spleens were compared between infected and non-infected mice following IL-10R neutralization (comparison group “IL-10R↓_early_/TMEV” vs. group “IL-10R↓_early_/mock”). Results revealed an increased percentage of CD19^+^ B cells (Fig 8) and decreased percentage of CD4^+^ T cells (Fig 8) within spleens following Ab treatment during the early TME phase (group “IL-10R↓_early_/TMEV”) at 14 dpi. Together with an upregulated Foxp3 mRNA expression (Table B in S1 File), relative numbers of Foxp3^+^ Treg were elevated (Fig 8) in animals receiving IL-10R Ab and TMEV (group “IL-10R↓_early_/TMEV”) compared to control animals (group “IL-10R↓_early_/mock”), representing a potential compensatory mechanism to inhibit overwhelming peripheral inflammatory responses and tissue damage, respectively. Spleen weights were significantly increased in IL-10R blocked mice following TMEV-infection at 22 dpi (Table B in S1 File). To further characterize the functional state of T cells, expression of activation markers CD69 and CD44 was investigated. CD69 and CD44 expression on both CD4^+^ T cells and CD8^+^ CTL were significantly enhanced in TMEV-infected animals (group “IL-10R↓_early_/TMEV”) compared to non-infected animals following cytokine receptor blockade (group “IL-10R↓_early_/mock”; Fig 8, Table B in S1 File).

Early administration of IL-10R blocking Ab led to an increased transcription of all investigated pro-inflammatory cytokines (IL-1α, IL-2, IL-5, IL-6, TNF, IFN-γ) and—probably in a compensatory manner—also of anti-inflammatory cytokines (IL-4, IL-10, TGF-β1) in TMEV-infected mice (group “IL-10R↓_early_/TMEV”) compared to non-infected animals (group “IL-10R↓_early_/mock”) at 14 and 22 dpi (Fig 8, Table B in S1 File). Worth mentioning, the highest cytokine transcription levels were measured in group “IL-10R↓_early_/TMEV” animals primarily at 14 dpi, associated with exacerbated colitis. Here, significantly higher levels of IFN-γ (p = 0.020) and statistical tendencies of increased levels of IL-2 (p = 0.066) and TNF (p = 0.066) were observed at 14 dpi compared to 22 dpi (data not shown), suggestive of a transient cytokine enhancement. Obtained data of experiment I are summarized in Tables C-E in S1 File.

**Fig 9:**
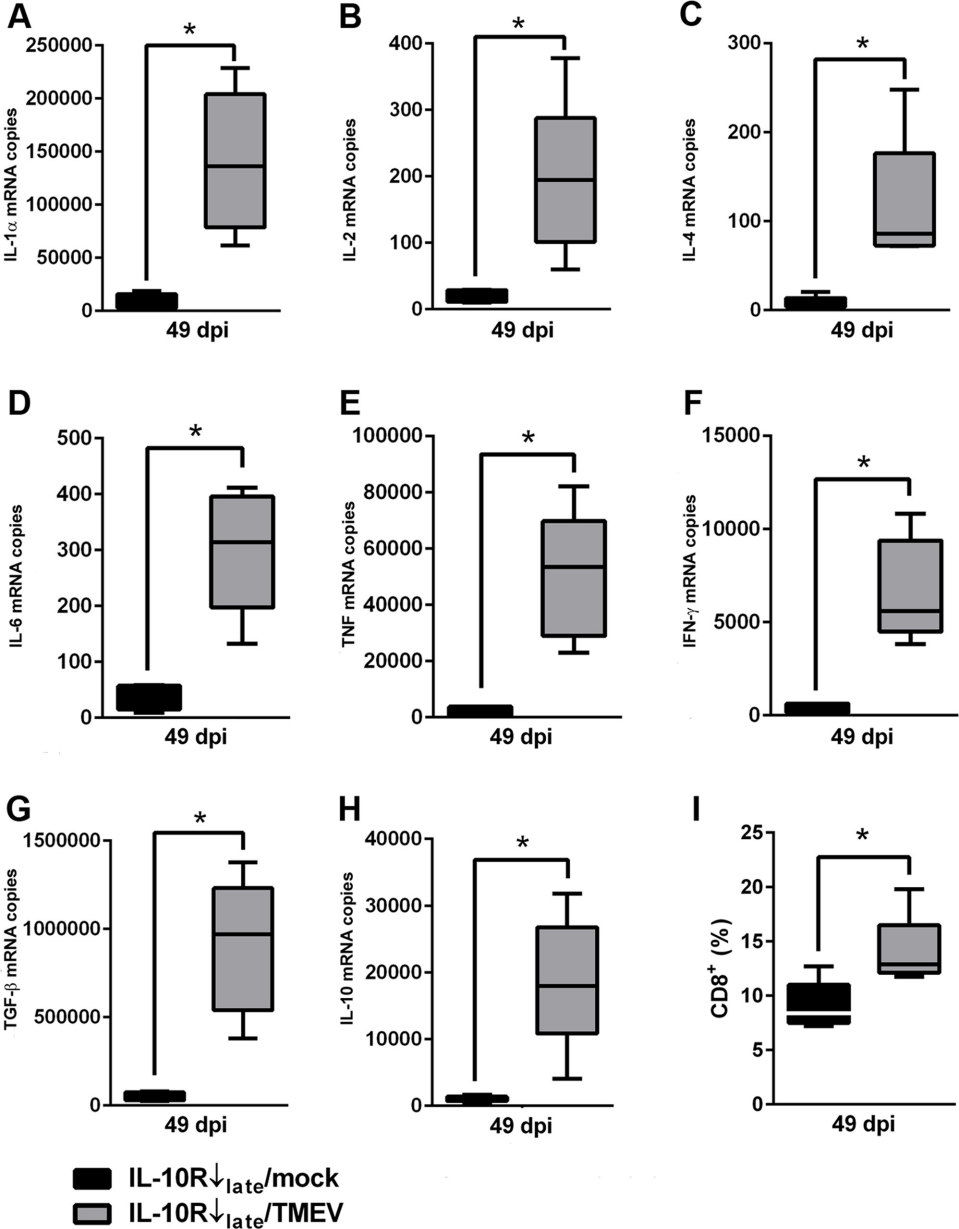
Effects of chronic Theiler’s murine encephalomyelitis virus (TMEV)-infection (*experiment II*) on cytokine expression and phenotypical changes in spleens of interleukin-10 receptor (IL-10R) neutralized SJL mice. (A-H) Significantly elevated mRNA levels of IL-1α, IL-2, IL-4, IL-6, TNF, IFN-γ, TGF-β and IL-10 in spleens of infected mice (group “IL-10R↓_late_/TMEV”) compared to non-infected animals following IL-10R antibody (Ab) treatment (group “IL-10R↓_late_/mock”). (I) Flow cytometry revealed a relative increase of CD8^+^ cells in the spleens of TMEV-infected mice with IL-10R Ab treatment at 49 dpi. For gating strategy see S2 Fig. IL-10R Ab treated mice without TMEV-infection (group “IL-10R↓_late_/mock”). IL-10R Ab treated mice with TMEV-infection (group “IL-10R↓_late_/TMEV”). Box and whisker plots display median, minimum and maximum values as well as upper and lower quartiles, 5 animals used in both groups, Wilcoxon rank-sum tests, *  =  p < 0.05.

In experiment II flow cytometric analyses was performed at 42 and 49 dpi and RT-qPCR was performed at 49 dpi in mice with Ab treatment during the chronic disease phase. A reduced percentage of CD4^+^ T cells together with an increased IFN-γ mRNA expression was observed in TMEV-infected mice following IL-10R blockade at 49 dpi (Table F in S1 File). Comparison between group “IL-10R↓_late_/TMEV” and group “IL-10R↓_late_/mock” revealed that IL-10R blockade during the late TME phase led to an elevated percentage of CD8^+^ CTL (Fig 9) but—in contrast to experiment I—induced no differences in the activation status (CD44, CD69) of CD4^+^ and CD8^+^ cells in infected mice (group “IL-10R↓_late_/TMEV”) compared to non-infected mice (group “IL-10R↓_late_/mock”; Table G in S1 File), indicative of disease phase-specific differences.

In analogy to experiment I, splenic cytokine expression in chronically infected mice was determined 14 days after the onset of Ab treatment (49 dpi). With the exception of IL-5, IL-10R neutralization at later time points significantly increased the mRNA-expression of all investigated pro- and anti-inflammatory cytokines at 49 dpi (Fig 9, Table F in S1 File) in infected mice. Obtained data of experiment II are listed in Tables A-G in S1 File.

In summary, results confirm the ability of acute CNS infection to cause prominent cytokine enhancement in IL-10R neutralized SJL mice, characteristic of a hyperactive immune state, which potentially worsened immune mediated colitis. The functional relevance is further shown by associated CD4^+^ T cell and CD8^+^ CTL activation (CD69 expression) together with an increased frequency of CD44^+^ memory T cells in acutely infected mice with anti-IL-10R Ab treatment (experiment I).

### Experiment III: interleukin-10 receptor blockade in non-infected animals

#### Interleukin-10 receptor blockade leads to the development of severe immune mediated colitis in SJL mice

**Fig 10:**
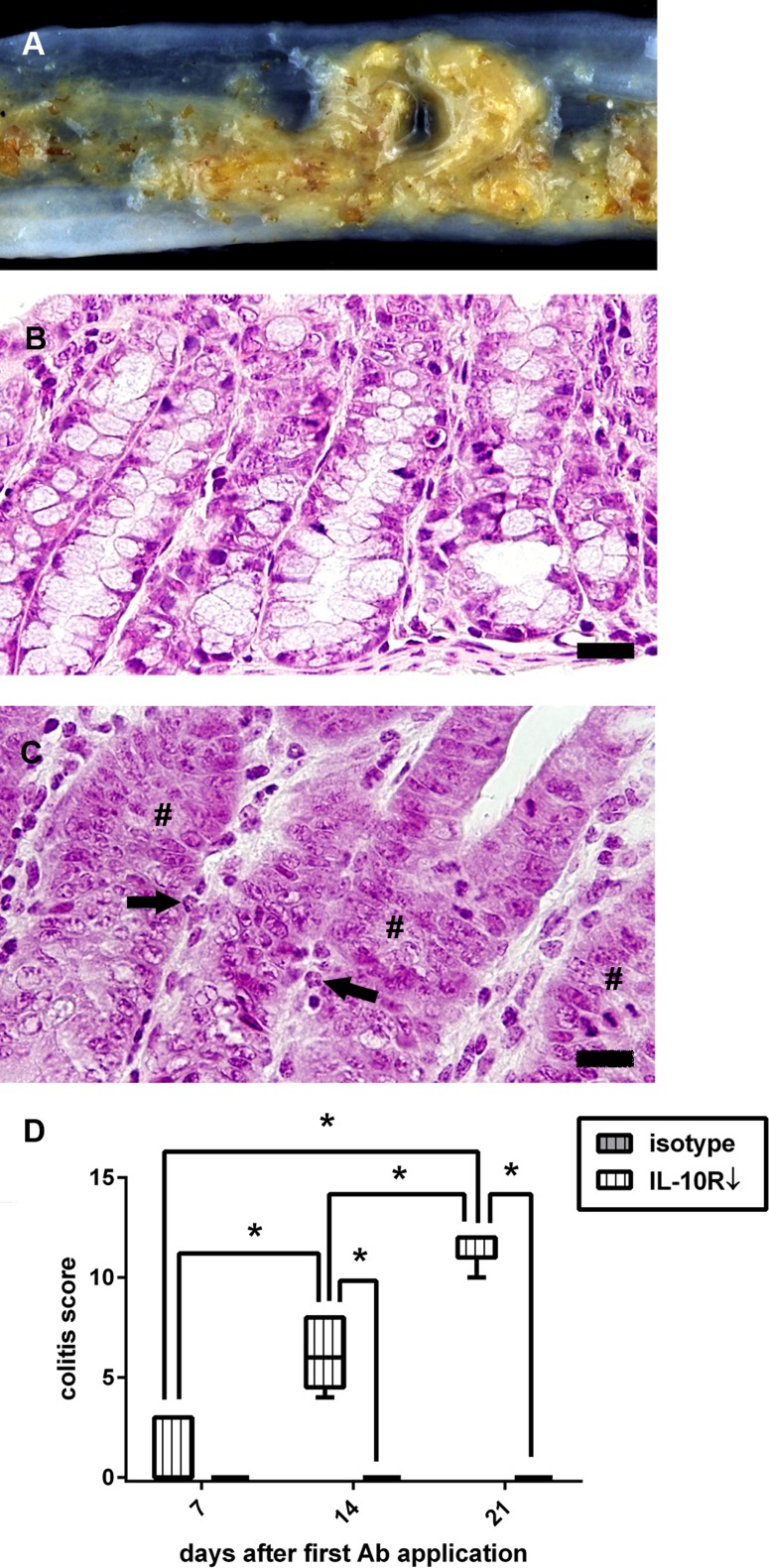
Enteric disease following interleukin-10 receptor (IL-10R) blockade in non-infected SJL mice. (A) Unformed feces and mucosal edema in an animal at 14 days after onset of IL-10R antibody (Ab) treatment. (B) Colon of a control animal (group “isotype”) at day 21 with normal mucosal architecture. (C) Colon of an animal receiving IL-10R Ab (group “IL-10R↓”) at the same time point. Note infiltration of neutrophilic granulocytes (arrows) and loss of goblet cells (#). B, C: H&E staining, bar = 20 μm. (D) IL-10R blockade causes progressive colitis compared to control animals. grey box with vertical lines = control animals (group “isotype”), white box with vertical lines = IL-10R blocked animals (group “IL-10R↓”). Box and whisker plot display median, minimum and maximum values as well as upper and lower quartiles, 5 animals used at all investigated time points, Wilcoxon rank-sum tests, *  =  p < 0.05.

**Fig 11:**
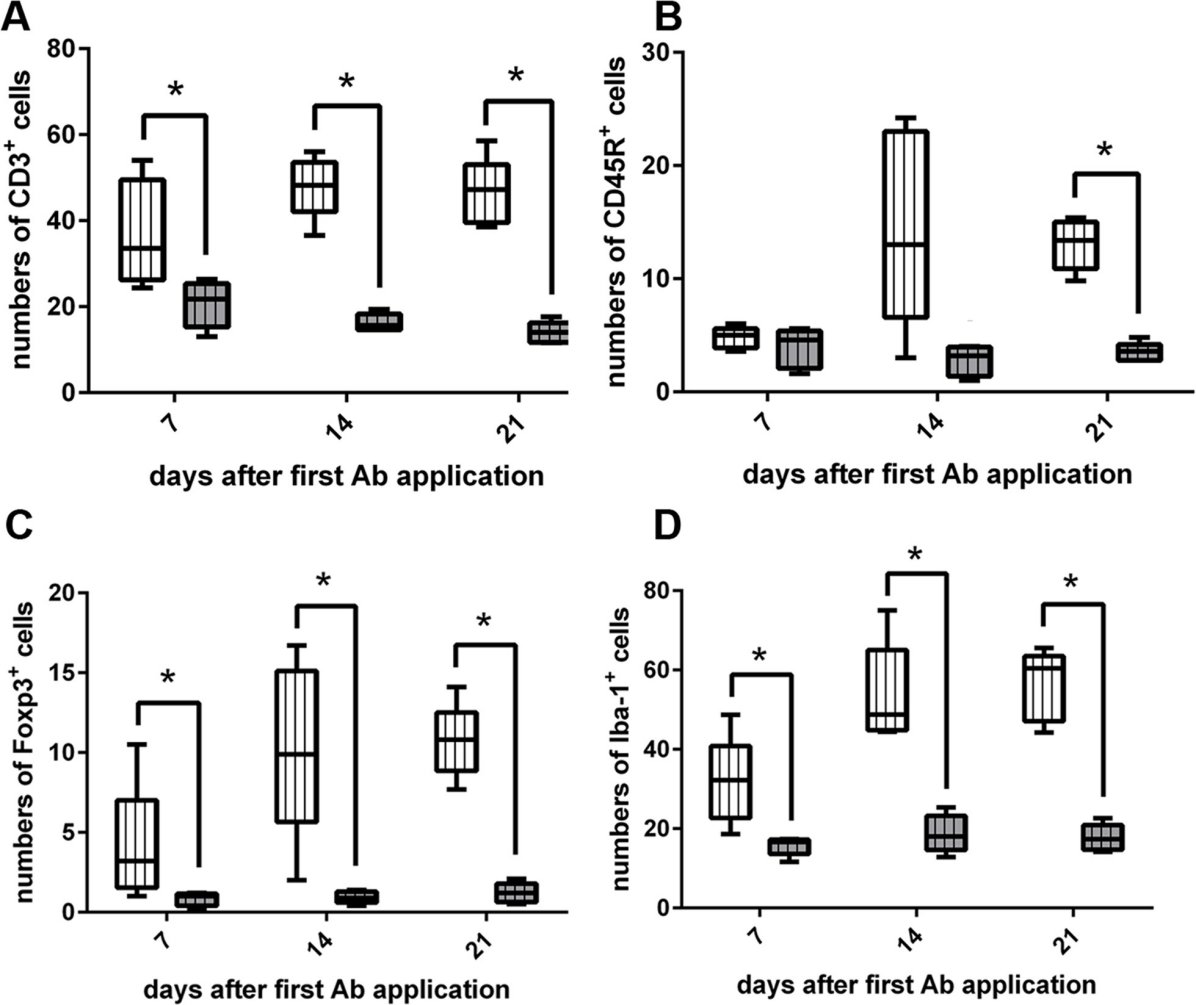
Leukocyte subsets in the colon of interleukin-10 receptor (IL-10R) neutralized SJL mice. (A) Application of IL-10R antibody (Ab) leads to increased numbers of CD3^+^ T cells in the colon at all investigated time points. (B). An increase of CD45R^+^ B cells can be observed at day 21 after the first Ab application. (C) Foxp3^+^ regulatory T cells and (D) Iba-1^+^ macrophages are elevated at 7, 14, and 21 dpi. grey box with vertical lines = control animals (group “isotype”), white box with vertical lines = IL-10R blocked animals (group “IL-10R↓”). Box and whisker plot display median, minimum and maximum values as well as upper and lower quartiles, 5 animals used at all investigated time points, Wilcoxon rank-sum tests, *  =  p < 0.05.

To characterize the effect upon peripheral organs of IL-10R blockade in SJL mice under non-infectious (steady-state) conditions, animals of experiment III received anti-IL-10R Ab (group “IL-10R↓”) or isotype control (group “isotype”) without additional TMEV-infection. Treatment successfully induced severe lymphohistiocytic to neutrophilic colitis with loss of goblet cells, crypt hyperplasia and crypt necrosis starting at day 7 (Fig 10). Histological changes in the intestine of these animals share common features with IBD lesions in IL-10 knockout mouse models [51]. Significant differences were present at different time points, showing a progressive disease course (Fig 10). IHC revealed that IL-10R blockade causes an early significant increase of CD3^+^ T cells, Foxp3^+^ Treg and Iba-1^+^ macrophages in the *lamina propria* of the colon at 7, 14, and 21 day (Fig 11). For CD45R^+^ B cells, a delayed significant infiltration of the colonic mucosa was found at day 21 after the first Ab treatment (Fig 11). Interestingly, intestinal lesions were not only restricted to the colon, since significant differences were observed between treated animals (group “IL-10R↓”) and controls (group “isotype”) also in the caecum at 21 dpi (Table H in S1 File). No histological changes of the small intestine were found at any time point. In addition, no inflammatory lesions were found in other tissues (see materials and methods), including the CNS. As expected, animals that received isotype control (group “isotype”) did not develop typhlocolitis (Fig 10). Additionally, intestinal inflammation was associated with significantly increased spleen weights of IL-10R blocked mice (group “IL-10R↓”) compared to control animals (group “isotype”) at 21 dpi. Obtained data of experiment III are summarized in Tables I and J in S1 File.

Results confirm the ability of IL-10R neutralization to breakdown gut immune homeostasis and induce immune-mediated colitis in SJL mice, resembling IBD.

#### Interleukin-10 receptor blockade in SJL mice is accompanied by decreased levels of CD4^+^ Foxp3^+^ regulatory T cells together with enhanced CD44 and CD69 expression on T cells in the spleen

**Fig 12:**
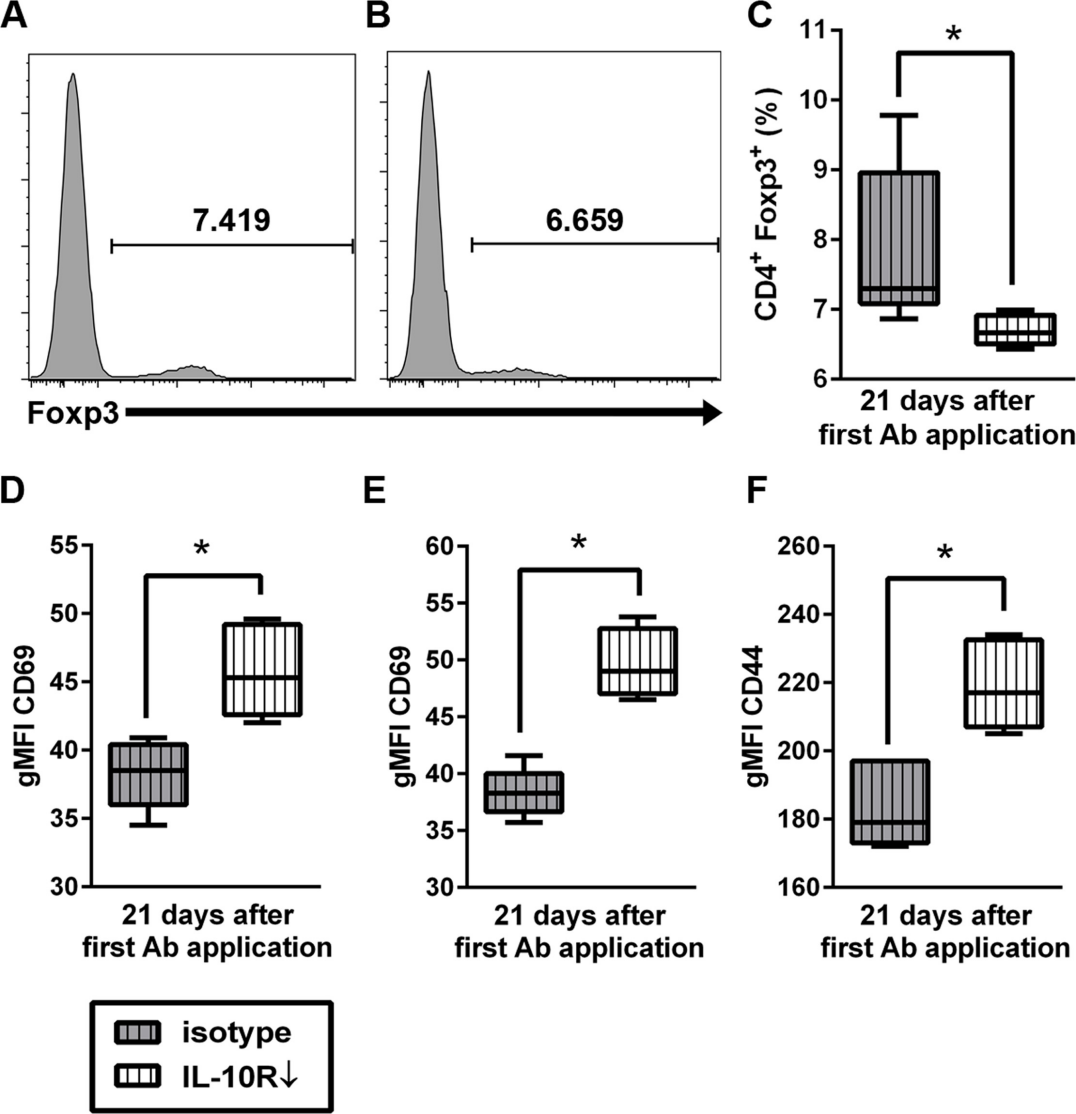
Effects of interleukin-10 receptor (IL-10R) blockade upon splenocytes of non-infected SJL mice. Histograms show higher relative numbers of CD4^+^Foxp3^+^ regulatory T cells in (A) control animals (group “isotype”) compared to (B) animals receiving anti-IL-10R antibody (IL-10R Ab; group “IL-10R↓”). For gating strategy see S3 Fig. (C) Relative decrease of splenic CD4^+^ Foxp3^+^ Treg in IL-10R blocked mice at day 21. Simultaneously, gMFI of CD69 gated on CD4^+^ cells (D) and CD8^+^ cells (E) were increased at day 21. (F) Similarly, an increased gMFI of CD44 gated on CD8^+^ cells was detected at day 21. grey box with vertical lines = control animals (group “isotype”), white box with vertical lines = IL-10R blocked animals (group “IL-10R↓”). Box and whisker plot display median, minimum and maximum values as well as upper and lower quartiles, 5 animals used in both groups, Wilcoxon rank-sum tests, *  =  p < 0.05.

In order to investigate effects upon peripheral immune responses associated with IL-10R blockade, phenotypical changes in the spleen were analyzed by flow cytometry at day 7, 14, and 21 of Ab treatment. Splenocytes from SJL mice with IL-10R blockade (group “IL-10R↓”) revealed significantly decreased percentages of CD4^**+**^ Foxp3^**+**^ Treg compared to control mice (group “isotype”) at day 21 after the first Ab application (Fig 12). Notably, relative numbers of CD3^**+**^ T cells, CD4^**+**^ T cells and CD8^**+**^ CTL remained unchanged, indicative of specific downregulation of Treg in Ab treated animals. Simultaneously, the gMFI of CD69 was significantly higher in CD4^**+**^ and CD8^**+**^ T cell subsets in IL-10R blocked mice (group “IL-10R↓”) and the gMFI of CD44, indicative of memory T cell differentiation, was elevated in CD8^**+**^ CTL of IL-10R-blocked animals (group “IL-10R↓”) compared to control mice (group “isotype”, Fig 8, Table H in S1 File). Obtained data of experiment III are summarized in Tables I and J in S1 File.

The finding of diminished CD4^+^ Foxp3^+^ Treg responses together with an enhanced activation and differentiation of T cells within the spleen supports the assumption, that IL-10R blockade disturbs peripheral immune tolerance, fostering systemic immunopathology.

## Discussion

IL-10 exhibits profound suppressive properties and thus critically controls the physiological balance of host immune responses [14, 64–66]. Besides immune-mediated colitis induced by dysregulated IL-10R signaling the study illustrates the complex interplay between neuroinflammation and peripheral immune responses in infectious disorders. The ability of CNS-restricted virus infection to aggravate systemic immunopathology via a hyperactive immune state is shown, representing an as yet undescribed phenomenon.

IL-10 is a pleiotropic cytokine which suppresses Th1 and Th17 responses and reduces antigen-presenting cell function, while enhancing Th2 responses, Treg expansion, B cell maturation and Ab production [67]. Genetic ablation of IL-10 or IL-10R leads to spontaneous colitis in mice, representing a reliable model for human IBD [26, 68]. Likewise, the present survey shows, that IL-10R neutralization induces early onset and severe enteric disease in SJL mice. Similar findings can be observed in very early-onset-IBD, a pediatric disorder caused by genetic defects affecting the IL-10R [20, 21, 23, 25]. Genome-wide association studies also revealed a central role of the IL-10 axis in adult IBD pathogenesis [69]. Furthermore, autoantibodies against IL-10 and IL-10R can be found in a subset of human IBD patients, however, their pathogenic relevance remains to be determined [70, 71]. Disturbed mucosal homeostasis in IL-10-deficient mouse models is accompanied by a loss of intestinal regulatory myeloid cells (CX_3_CR1^high^CD11b^+^CD11c^+^) and Treg (CD4^+^Foxp3^+^) leading to aberrant Th1/Th17-mediated responses towards commensal gut microbiota and dietary Ag [72]. Noteworthy, genetic IL-10R deficits lead to a pro-inflammatory phenotype of regulatory CX_3_CR1^high^ macrophages and development of colitis in mice [66]. In the present study also reduced numbers of CD4^+^Foxp3^+^ Treg together with increased numbers of CTL in the spleen indicates unbalanced immune responses, which may contribute to progressive intestinal inflammation. IHC revealed an initial infiltration of predominately CD3^+^ T cells and Iba1^+^ macrophages with a delayed CD45R^+^ B cell infiltration in the colon of IL-10R neutralized mice, suggestive of a T cell-mediated immunopathology. Dominating Th1- and/or Th17-mediated cellular immune responses can be observed also in Crohn`s disease, while Th2-mediated humoral responses are supposed to account for ulcerative colitis [73, 74]. Similar to human IBD, a seemingly paradox increased mucosal infiltration of Foxp3^+^ T cells is associated with colitis in IL-10R blocked mice [75–77]. Since Treg are critical for preventing intestinal inflammation, mucosal Foxp3^+^ T cells are unable to control gut immune homeostasis in the present study, either by an insufficient amount or cellular dysfunction, as described for immune mediated colitis in human patients [76–80]. The recruitment and sequestration of Foxp3^+^ T cells in the colon might contribute to the reduced frequency of splenic Treg in IL-10R neutralized mice observed by flow cytometry.

Susceptibility to developing intestinal inflammation in most models of experimental colitis differs among inbred mouse strains. In a previously established inducible IBD model, C57BL/6 mice infected with the pathobiont *Helicobacter hepaticus*, but not animals lacking these bacteria, responded to Ab-mediated IL-10R neutralization by colitis development [81]. Using SJL mice, which are prone to develop Th1-driven disorders [42, 43], we were able to induce similar intestinal lesions without an additional bacterial trigger. The predominately detrimental response of SJL mice to IL-10 signaling defects is also exemplified by therapeutic effects of Ab-mediated IL-10 or IL-10R blockade observed in WNV-infected C57BL/6 mice and LCMV-infected BALB/c mice [29–32].

Most strikingly, acute TMEV-infection has the capacity to transiently exacerbate enteric disease in IL-10R blocked mice. Here, the lack of a detectable influence of chronic TME upon enteritis severity indicates a disease phase-specific effect. Present data show that CNS infection causes a systemic hyperactive immune state following IL-10R blockade with up-regulation of pro- and anti-inflammatory cytokines. Although proof of causality is often missing, infections are supposed to trigger autoimmune disorders, such as MS, type 1 diabetes, Guillian-Barré syndrome and systemic lupus erythematosus, by molecular mimicry, epitope spreading, release of cryptic Ag or bystander activation, respectively [82, 83]. Similarly, participation of viruses (e.g. measles virus, mumps virus, cytomegalovirus, Epstein-Barr virus) in the initiation or exacerbation of IBD in human patients is currently under discussion [84, 85]. Our data provide evidence that extraintestinal infection can enhance enteric disease. Recently, similar effects have been described in experimental respiratory influenza virus infection, where intestinal injury is caused by lung-derived virus-specific CD4^+^ effector T cells, which destroy intestinal homeostasis and promote Th17 cell polarization [86]. Moreover, during the onset of EAE intestinal barrier dysfunction together with disturbed peripheral immune homeostasis probably caused by circulating myelin-specific encephalitogenic T cells has been described [87]. Thus, exacerbated colitis following TMEV-infection might be caused by an unspecific (bystander) stimulation of colitogenic T cells directed against different dietary antigens and/or gut microbiota due to an activation of peripheral immune responses. Other explanations include molecular mimicry between released CNS antigens and intestinal antigens and subsequent cross-reactivity of autoimmune lymphocytes, as discussed for the association (co-morbidity) between MS and IBD [88, 89]. In addition, stress increases intestinal permeability and exacerbates enteritis in rats, suggestive of brain-gut interaction [90, 91]. Although the basic mechanisms remain undetermined, also a correlation between psychological disorders and the prevalence and course of Crohn’s disease has been reported [92–94]. Conversely, intestinal barrier damage and gut microbiota are supposed to trigger systemic and CNS autoimmunity [89, 95, 96], which might have contributed to the observed mild increased CD3^+^ T cell infiltration in the spinal cord during chronic TME (see below). Increased intestinal permeability often referred to as “leaky gut” syndrome can also be observed in MS and systemic autoimmune diseases, such as type I diabetes [97]. Interestingly, concurrent IBD has been observed in a subset of MS patients [88, 98]. The cause of this co-morbidity is still unclear, but genetic factors that predispose individuals to develop immune mediated disorders are assumed [99].

During the acute TME phase, hyperactive immune state with enhanced cytokine expression following IL-10R neutralization is accompanied by an elevated frequency of CD4^+^CD69^+^ and CD8^+^CD69^+^ activated T cells and increased percentage of CD8^+^CD44^+^ and CD4^+^CD44^+^ memory T cells (14 dpi). Enhanced CD8-mediated cytotoxicity can contribute to intestinal inflammation in murine colitis models [100, 101]. Moreover, comparable with the present findings, an increased activation and memory differentiation of circulating CD8^+^ CTL is associated with an aggressive disease course including frequent clinical relapses in human IBD patients [102]. Furthermore, intestinal tract damage is associated with systemic CD4^+^ and CD8^+^ T cell activation [103]. Using DNA microarray analyses, we previously observed an activation of lymphoid organs (cervical LN, spleen) of SJL mice during early TME (14 dpi), followed by transcriptional silencing during the chronic demyelinating phase [53]. Thus, the transient nature of peripheral immune activation in the TME model might explain the observed temporary worsening of IL-10R blockade-mediated enteritis during the acute infection and disease phase-dependent alterations of peripheral immune responses, respectively. The increased frequency of CD4^+^Foxp^+^ Treg, together with an enhanced mRNA expression of Foxp3 and modulatory cytokines, such as IL-10 and TGF-β, might represent compensatory attempts to sustain peripheral immune homeostasis and dampen overwhelming immune responses following IL-10R neutralization and concurrent acute TMEV infection [17, 75–77]. Expansion of Foxp3 Treg has been shown to efficiently reduce antiviral immunity in TMEV-infected SJL mice [104]. However, probably due to the limited Treg recruitment to the CNS, as described by Foxp3-specific immunohistochemistry and RT-qPCR, no treatment effects upon viral load or neuropathology were found in the present study.

A mild increased CD3^+^ T cells influx together with reduced IL-6 expression was observed in the infected spinal cord following IL-10R blockade during the chronic TME phase. Similarly, enhanced TMEV-specific CD4^+^ and CD8^+^ T cell responses in the CNS are associated with decreased IL-10 production in CD11b^+^Ly6c^+^ depleted mice [105] and in MS patients a leukocyte disability to produce IL-10 is supposed to foster CNS disease progression [106]. The inhibitory effect of IL-10 upon neuroinflammation is also illustrated by IL-10 or IL-10R blockade in EAE [107, 108]. However, in contrast to the EAE model no overt changes upon spinal cord pathology were observed in TMEV-infected mice following Ab treatment in the present experiments. IL-6 represents a pleotropic cytokine exhibiting detrimental and neuroprotective effects in different CNS disorders [109]. A complex and probably ambivalent function of IL-6 can also be observed in the TME model. For instance, treatment with recombinant IL-6 reduces spinal cord demyelination in susceptible mice infected with the Daniel`s strain of TMEV [110]. Moreover, exogenous IL-6 inhibits TMEV replication in cultured macrophages [111]. By contrast, studies using the BeAn-TMEV strain have shown that excessive IL-6 promotes pathogenic Th17 responses and viral persistence by preventing apoptosis of virus-infected cells [5, 112]. IL-6, in interplay with other cytokines, also contributes to T cell exhaustion and inadequate viral elimination in TME [113, 114]. However, sole IL-6 reduction following IL-10R neutralization is apparently insufficient to alter local CNS inflammatory responses in the present study. In addition, CNS virus load remained unchanged, indicative of an unaffected antiviral immunity. Possible explanations for the limited effect of anti-IL-10R treatment in TME include (i) autonomous immune responses triggered by virus persistence in the spinal cord, (ii) an inability of the anti-IL-10R Ab to penetrate the blood spinal cord barrier and reach sufficient CNS levels, and/or (iii) a preferential recruitment of inflammatory cells to the intestine with peripheral sequestration. In TMEV-infected SJL mice local Ag presentation and plasma cell differentiation in the spinal cord together with an intact blood spinal cord barrier are supposed to cause a CNS-restricted inflammation [115–117]. Similarly, closure of the blood brain barrier, isolating the CNS from peripheral lymphoid organs, is supposed to play a pivotal role in therapy failure in chronic MS patients [118–122]. In general, CNS uptake of therapeutic antibodies is limited by an intact neurovascular endothelium [123]. Thus, prominent blood brain barrier damage and permeability present in experimental WNV- or LCMV-infection explains the restoration of antiviral immunity in the CNS and beneficial effects of anti-IL-10/IL10-R treatment in these infection models in contrast to TME [30, 31, 124, 125]. Detrimental effects of IL-10R blockade in TME might be circumvented by adequate Ab delivery systems across the blood spinal cord barrier to guarantee sufficient CNS Ab levels and to reduce systemic adverse effects [123, 126–128].

## Conclusions

In summary, unlike other CNS infectious models pharmacological blockade of IL-10 signaling in TME causes severe systemic immunopathology in mouse strains with a susceptible genetic background. Since IL-10 is currently discussed as target for novel therapeutic approaches in chronic viral diseases, identifing critical factors that regulate IL-10 in different organ compartments is crucial for understanding how IL-10 restores protective immunity, especially in patients with genetic predisposition to develop immune mediates disorders [129].

## Supporting Information

S1 Fig Gating strategy for flow cytometric analyses shown in Fig 8. (A) Gating of splenocytes from control animals receiving mock-infection and intraperitoneal interleukin-10 receptor (IL-10R) antibody (Ab) application in the early infection phase (group “IL-10R↓_early_/mock”). (B) Gating of splenocytes from animals receiving Theiler’s murine encephalomyelitis virus (TMEV)-infection and IL-10R Ab (group “IL-10R↓_early_/TMEV”). Cells were first gated for granularity, size and singlets followed by Live/Dead staining for exclusion of death cells. Living cells were tested for surface expression of CD4 and CD8 and viable CD4 expressing subsets were gated for expression of Foxp3. The gMFI for CD69 and CD44 was calculated for CD4 and CD8 expressing cells separately by use of FlowJo software.
(TIF)

S2 Fig Gating strategy for flow cytometric analyses shown in Fig 9. (A) Gating of splenocytes from control animals receiving mock-infection and intraperitoneal application of interleukin-10 receptor (IL-10R) antibody (Ab; group “IL-10R↓_late_/mock”). (B) Gating of splenocytes from animals receiving Theiler’s murine encephalomyelitis virus (TMEV)-infection and IL-10R Ab (group “IL-10R↓_late_/TMEV”). Cells were first gated for granularity, size and singlets followed by Live/Dead staining for exclusion of death cells. Living cells were tested for surface expression of CD4 and CD8.
(TIF)

S3 Fig Gating strategy for flow cytometric analyses shown in Fig 12. (A) Gating of splenocytes from control animals (group “isotype”). (B) Gating of splenocytes from animals receiving interleukin-10 receptor antibody (group “IL-10R↓”). Cells were first gated for granularity, size and singlets followed by Live/Dead staining for exclusion of death cells. Living cells were tested for surface expression of CD3 and CD19. CD4 and CD8 surface expression was gated on CD3 expressing viable cells and Foxp3 expression was subsequently analyzed in the CD4^+^ T cell subset.
(TIF)

S4 Fig Lack of Theiler’s murine encephalomyelitis virus (TMEV)-antigen and -RNA in the colon of infected animals. (A) TMEV-specific immunohistochemistry revealed virus antigen (arrows) in the white and adjacent grey matter of the spinal cord at 49 dpi (positive control). (B) Similarly, TMEV-specific *in situ* hybridization revealed presence of virus RNA in the spinal cord. In contrast, no TMEV-specific antigen (C) or TMEV-specific RNA (D) were detected in the colon of animals. A,C: Immunohistochemistry, 400x, B,D: *in situ* hybridization, 200x.
(TIF)

S1 File Statistical analysis and median, minimum and maximum values of performed experiments. **Table A. Summary of statistical analyses of *experiment I*: effects of IL-10R blockade during acute Theiler’s murine encephalomyelitis.** arrows **=** significant up (↑)**-** or downregulation (↓) in TMEV-infected SJL mice receiving anti-IL-10R Ab compared with mice receiving IgG1 specific isotype control in the acute phase of the disease; administration of Ab or isotype control, respectively, was performed at 0, 7, 14 and 21 dpi; * bold p-values **=** significant difference between both groups, p ≤ 0.05, determined by Wilcoxon´s rank sum-tests. TMEV **=** Theiler´s murine encephalomyelitis virus; dpi **=** days post infection; IL **=** interleukin; TNF **=** tumor necrosis factor; TGF **=** transmissible growth factor; INF **=** interferon; Foxp3 **=** forkhead box P3 protein; MBP **=** myelin basic protein; np-NF **=** non-phosphorylated neurofilament; gMFI **=** geometric mean of fluorescence intensity; ND **=** not determined. **Table B. Summary of statistical analyses of *experiment I*: effects of acute Theiler’s murine encephalomyelitis virus infection upon IL-10R blockade.** arrows **=** significant up(↑)**-** or downregulation (↓) in TMEV-infected SJL mice receiving anti-IL-10R Ab compared to non-infected mice receiving anti-IL-10R Ab during the acute infection phase (0, 7, 14 and 21 dpi); * bold p-values display significant difference between both groups (p ≤ 0.05) determined by Wilcoxon´s rank sum-tests. TMEV **=** Theiler´s murine encephalomyelitis virus; dpi **=** days post infection; IL **=** interleukin; TNF **=** tumor necrosis factor; TGF **=** transmissible growth factor; INF **=** interferon; Foxp3 **=** forkhead box P3 protein; MBP **=** myelin basic protein; np-NF **=** non-phosphorylated neurofilament; gMFI **=** geometric mean of fluorescence intensity; ND **=** not determined. **Table C. Summary of data obtained from animals receiving TMEV-infection and intraperitoneal application of anti-IL-10R antibody (group “IL10R↓early/TMEV” [experiment I] and group “IL10R↓late/TMEV” [experiment II]).** TMEV **=** Theiler´s murine encephalomyelitis virus; dpi **=** days post infection; IL **=** interleukin; TNF **=** tumor necrosis factor; TGF **=** transmissible growth factor; INF **=** interferon; Foxp3 **=** forkhead box P3 protein; MBP **=** myelin basic protein; np-NF **=** non-phosphorylated neurofilament; gMFI **=** geometric mean of fluorescence intensity; ND **=** not determined; units: 1 **=** rounds per minute; 2 **=** points (semiquantitative scoring); 3 **=** gram; 4 **=** number of labelled cells in the spinal cord; 5 **=** percentage [%] of MBP-unstained (demyelinated) area; 6 **=** number of labelled axons in the spinal cord; 7 **=** copy numbers; 8 **=** percentage [%] of labelled cells. **Table D. Summary of data obtained from animals receiving TMEV-infection and intraperitoneal application of IgG1-specific isotype control (group “isotypeearly/TMEV” [experiment I] and group “isotypelate/TMEV” [experiment II]).** TMEV **=** Theiler´s murine encephalomyelitis virus; dpi **=** days post infection; IL **=** interleukin; TNF **=** tumor necrosis factor; TGF **=** transmissible growth factor; INF **=** interferon; Foxp3 **=** forkhead box P3 protein; MBP **=** myelin basic protein; np-NF **=** non-phosphorylated neurofilament; gMFI **=** geometric mean of fluorescence intensity; ND **=** not determined; units: 1 **=** rounds per minute; 2 **=** points (semiquantitative scoring); 3 **=** gram; 4 **=** number of labelled cells in the cord; 5 **=** percentage [%] of MBP-unstained (demyelinated) area; 6 **=** number of labelled axons in the spinal cord; 7 **=** copy numbers; 8 **=** percentage [%] of labelled cells. **Table E. Summary of data obtained from animals receiving mock-infection and intraperitoneal application of anti-IL-10R antibody (group “isotypeearly/mock” [experiment I] and group “isotypelate/mock” [experiment II]).** TMEV **=** Theiler´s murine encephalomyelitis virus; dpi **=** days post infection; IL **=** interleukin; TNF **=** tumor necrosis factor; TGF **=** transmissible growth factor; INF **=** interferon; Foxp3 **=** forkhead box P3 protein; MBP **=** myelin basic protein; np-NF **=** non-phosphorylated neurofilament; gMFI **=** geometric mean of fluorescence intensity;ND **=** not determined; units: 1 **=** rounds per minute; 2 **=** points (semiquantitative scoring); 3 **=** gram; 4 **=** number of labelled cells/axons in the spinal cord; 5 **=** percentage [%] of MBP-unstained (demyelinated) area; 6 **=** copy numbers; 7 **=** percentage [%] of labelled cells. **Table F. Summary of statistical analyses of *experiment II*: effects of IL-10R blockade during chronic Theiler’s murine encephalomyelitis.** arrow **=** significant up(↑)**-** or downregulation (↓) in TMEV-infected SJL mice receiving anti-IL-10R Ab compared with mice receiving IgG1 specific isotype control in the chronic phase of the disease; administration of Ab or isotype control respectively was performed at 35 and 42 dpi; * bold p-values **=** significant difference between both groups, p ≤ 0.05, determined by Wilcoxon´s rank sum-tests. TMEV **=** Theiler´s murine encephalomyelitis virus; dpi **=** days post infection; IL **=** interleukin; TNF **=** tumor necrosis factor; TGF **=** transmissible growth factor; INF **=** interferon; Foxp3 **=** forkhead box P3 protein; MBP **=** myelin basic protein; np-NF **=** non-phosphorylated neurofilament; gMFI **=** geometric mean of fluorescence intensity; ND **=** not determined. **Table G. Summary of statistical analyses of *experiment II*: effects of chronic Theiler’s murine encephalomyelitis virus infection upon IL-10R blockade.** arrows **=** significant up-regulation (↑) or down-regulation (↓) in infected SJL mice receiving IL-10R Ab compared to non-infected mice receiving anti-IL-10R Ab during the chronic infection phase (35 and 42 dpi); * bold p-values display significant differences between both groups (p ≤ 0.05) determined by Wilcoxon´s rank sum-tests. TMEV **=** Theiler´s murine encephalomyelitis virus; dpi **=** days post infection; Foxp3 **=** forkhead box P3 protein; MBP **=** myelin basic protein; np-NF **=** non-phosphorylated neurofilament; gMFI **=** geometric mean of fluorescence intensity. **Table H. Summary of statistical analyses of *experiment III*: effects of IL-10R blockade in non-infected animals.** arrow **=** significant up(↑)**-** or downregulation (↓) in SJL mice receiving anti-IL-10R Ab compared with mice receiving IgG1 specific isotype only; administration of Ab or isotype control, respectively, was performed at day 0, 7 and 14; * bold p-values **=** significant difference between both groups, p ≤ 0.05, determined by Wilcoxon´s rank sum-tests. Foxp3 **=** Iba-1 **=** ionized calcium binding adaptor molecule 1; forkhead box P3 protein; gMFI **=** geometric mean of fluorescence intensity. **Table I. Summary of data obtained from non-infected animals receiving intraperitoneal application of anti-IL10R Ab (group “IL10R↓”; experiment III).** * given are median values (minimum and maximum); gMFI **=** geometric mean of fluorescence intensity; units: 1 **=** points (semiquantitative scoring); 2 **=** number of labelled cells in the lamina propria per high power field, 3 **=** percentage [%] of labelled cells. **Table J. Summary of data obtained from non-infected animals receiving intraperitoneal application of IgG1-specific isotype control (group “isotype”; experiment III).** * given are median values (minimum and maximum); gMFI **=** geometric mean of fluorescence intensity; units: 1 **=** points (semiquantitative scoring); 2 **=** number of labelled cells in the lamina propria per high power field; 3 **=** percentage [%] of labelled cells.
(DOC)

## Abbreviations

CTL: cytotoxic T lymphocytes
dpi: days post infection
EAE: experimental autoimmune encephalomyelitis
gMFI: geometric mean of fluorescence intensity
H&E: hematoxylin and eosin
IFN-γ: interferon-γ
IgG1: immunoglobulin G1
IHC: immunohistochemistry
IL-10: Interleukin-10
IL-10R: Interleukin-10 receptor
Iba-1: ionized calcium binding adaptor molecule 1
i.p.: intraperitoneal
ISH: in situ hybridization
LCMV: lymphocytic choriomeningitis virus
LN: lymph node
mRNA: messenger RNA
MS: multiple sclerosis
MBP: myelin basic protein
np-NF: non-phosphorylated neurofilament protein
PVI: perivascular infiltrates
qPCR: real-time quantitative polymerase chain reaction
STAT3: signal transducer and activator of transcription 3
Th cells: T helper cells
TME: Theiler’s murine encephalomyelitis
TMEV: Theiler´s murine encephalomyelitis virus
TGF-β: transforming growth factor-β
Treg: regulatory T cells
TNF: tumor necrosis factor
WNV: West Nile Virus
