# Supplementary figures and images for: Viral Infection of the Central Nervous System Exacerbates Interleukin-10 Receptor Deficiency-Mediated Colitis in SJL Mice
Source: PLoS One. 2016 Sep 9;11(9):e0161883. doi: 10.1371/journal.pone.0161883 (PMC5017624; doi:10.1371/journal.pone.0161883)

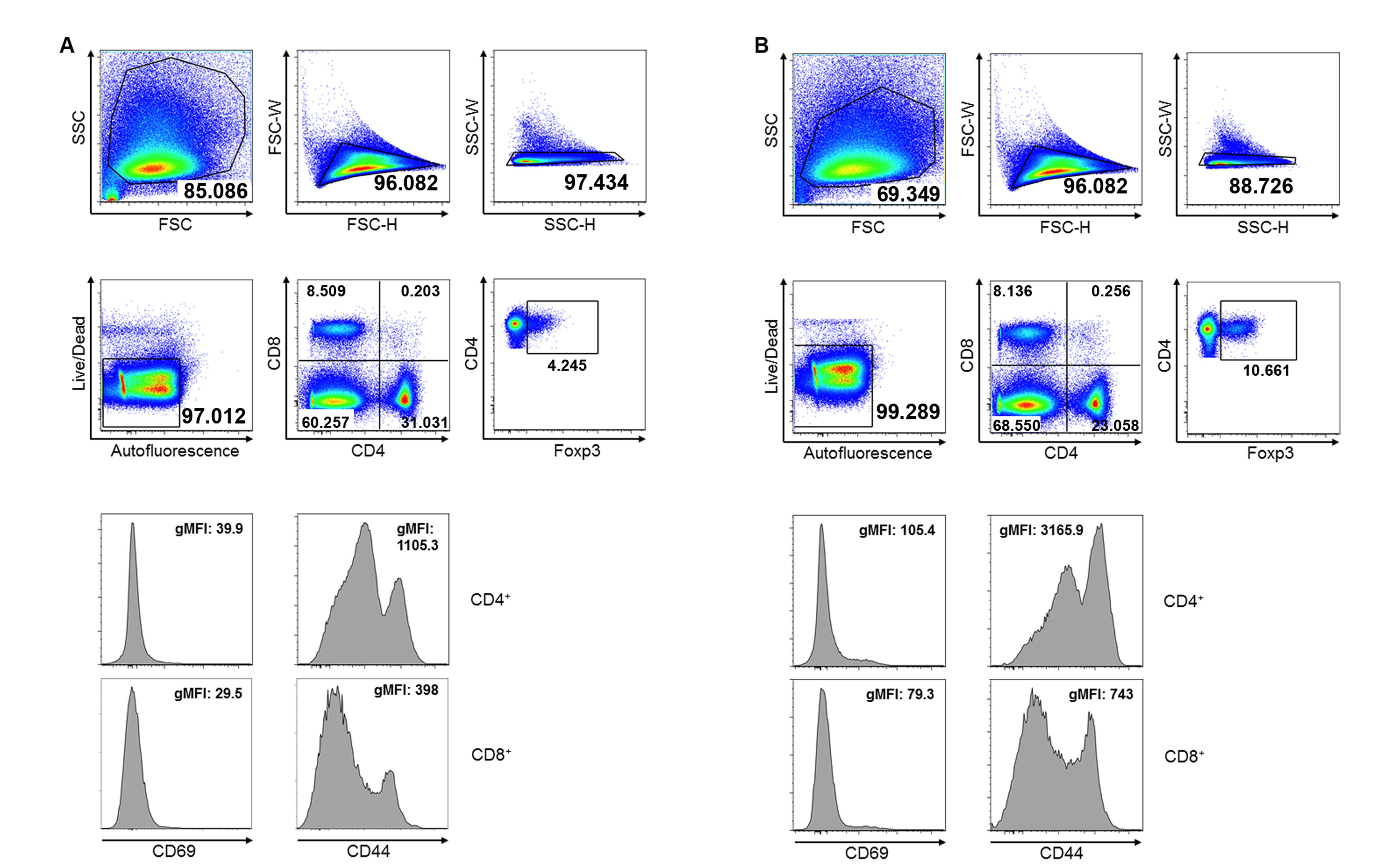

Supplement: S1 Fig — (A) Gating of splenocytes from control animals receiving mock-infection and intraperitoneal interleukin-10 receptor (IL-10R) antibody (Ab) application in the early infection phase (group “IL-10R↓early/mock”). (B) Gating of splenocytes from animals receiving Theiler’s murine encephalomyelitis virus (TMEV)-infection and IL-10R Ab (group “IL-10R↓early/TMEV”). Cells were first gated for granularity, size and singlets followed by Live/Dead staining for exclusion of death cells. Living cells were tested for surface expression of CD4 and CD8 and viable CD4 expressing subsets were gated for expression of Foxp3. The gMFI for CD69 and CD44 was calculated for CD4 and CD8 expressing cells separately by use of FlowJo software. (TIF) [file pone.0161883.s001.tif]

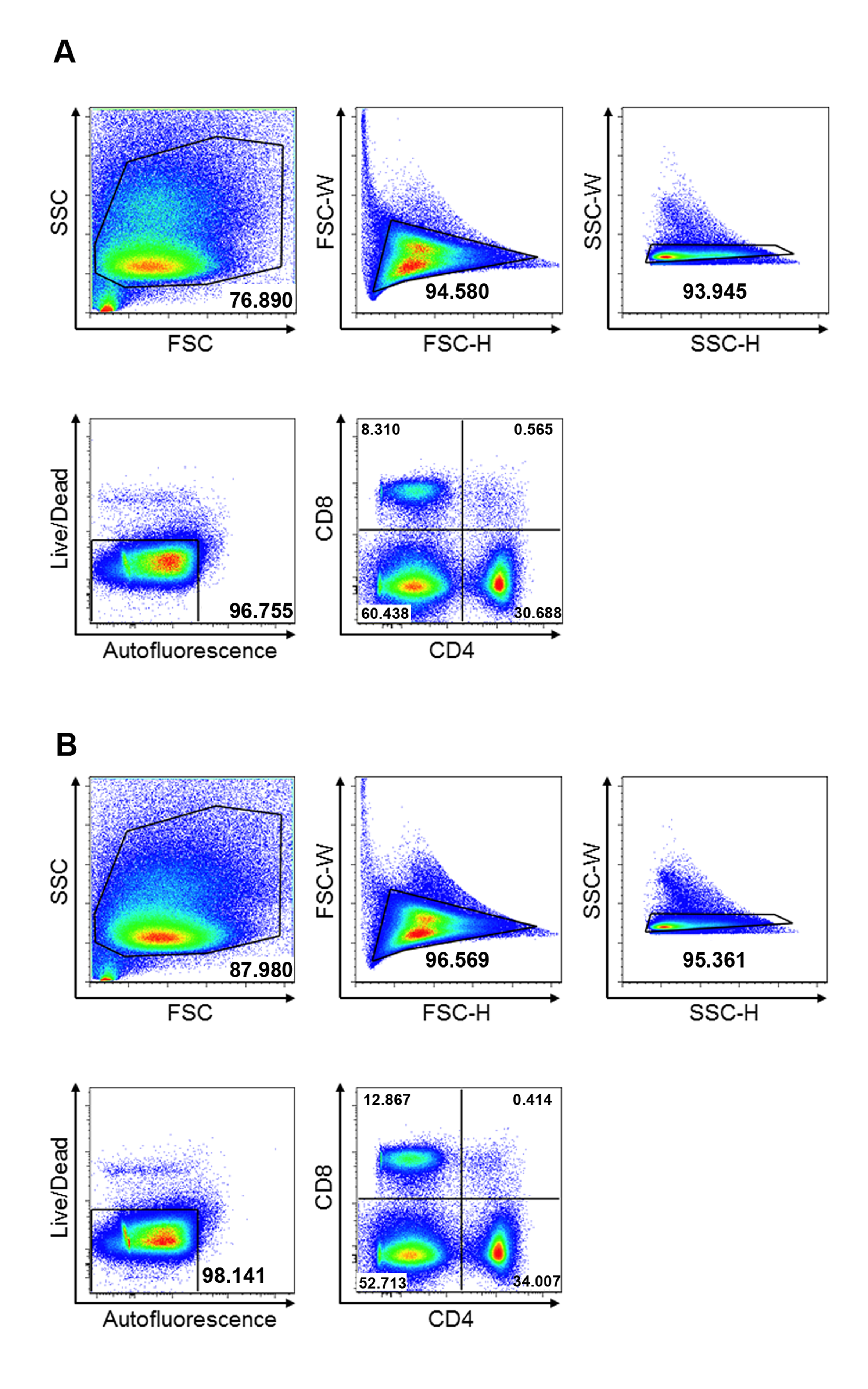

Supplement: S2 Fig — (A) Gating of splenocytes from control animals receiving mock-infection and intraperitoneal application of interleukin-10 receptor (IL-10R) antibody (Ab; group “IL-10R↓late/mock”). (B) Gating of splenocytes from animals receiving Theiler’s murine encephalomyelitis virus (TMEV)-infection and IL-10R Ab (group “IL-10R↓late/TMEV”). Cells were first gated for granularity, size and singlets followed by Live/Dead staining for exclusion of death cells. Living cells were tested for surface expression of CD4 and CD8. (TIF) [file pone.0161883.s002.tif]

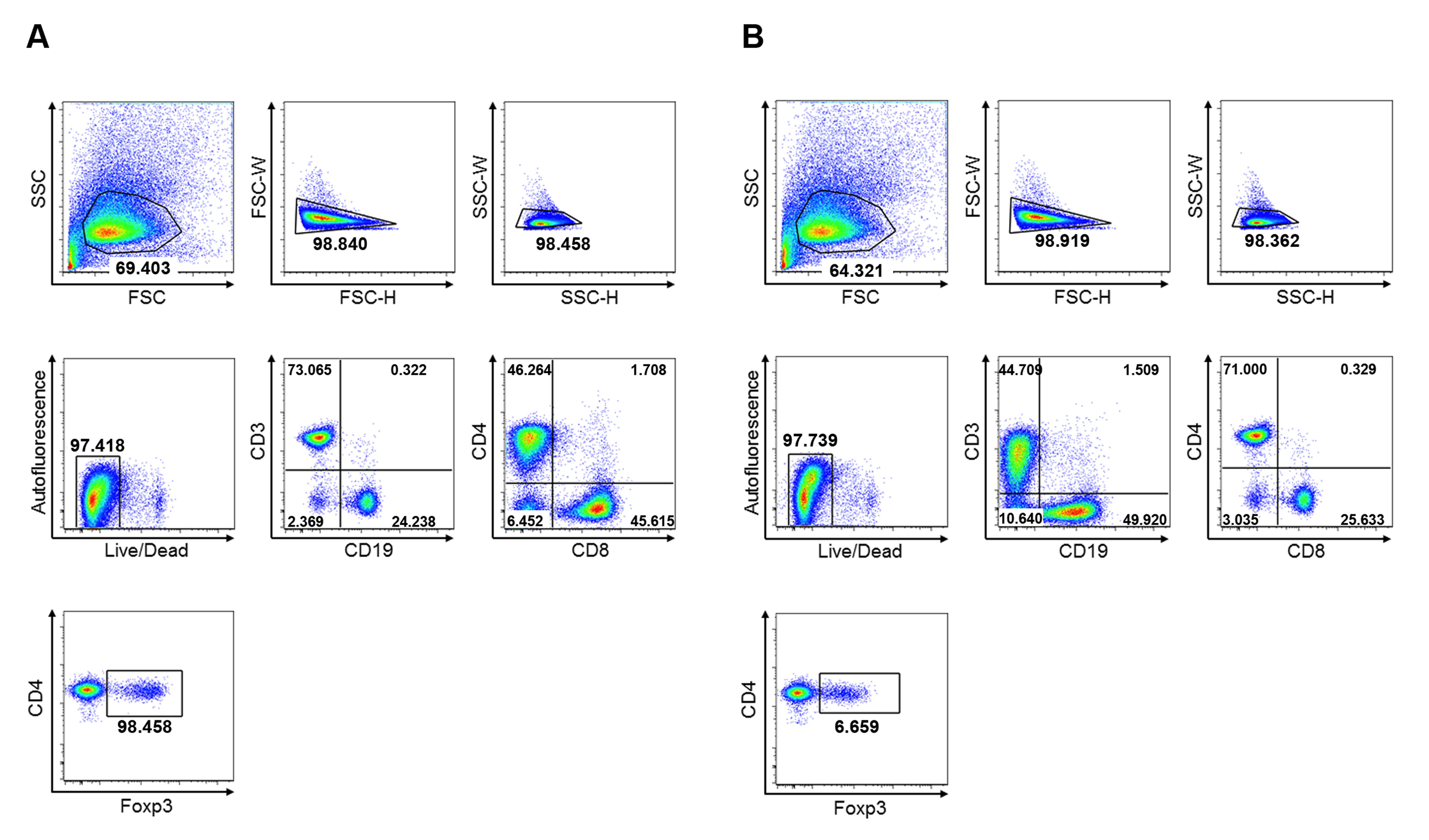

Supplement: S3 Fig — (A) Gating of splenocytes from control animals (group “isotype”). (B) Gating of splenocytes from animals receiving interleukin-10 receptor antibody (group “IL-10R↓”). Cells were first gated for granularity, size and singlets followed by Live/Dead staining for exclusion of death cells. Living cells were tested for surface expression of CD3 and CD19. CD4 and CD8 surface expression was gated on CD3 expressing viable cells and Foxp3 expression was subsequently analyzed in the CD4+ T cell subset. (TIF) [file pone.0161883.s003.tif]

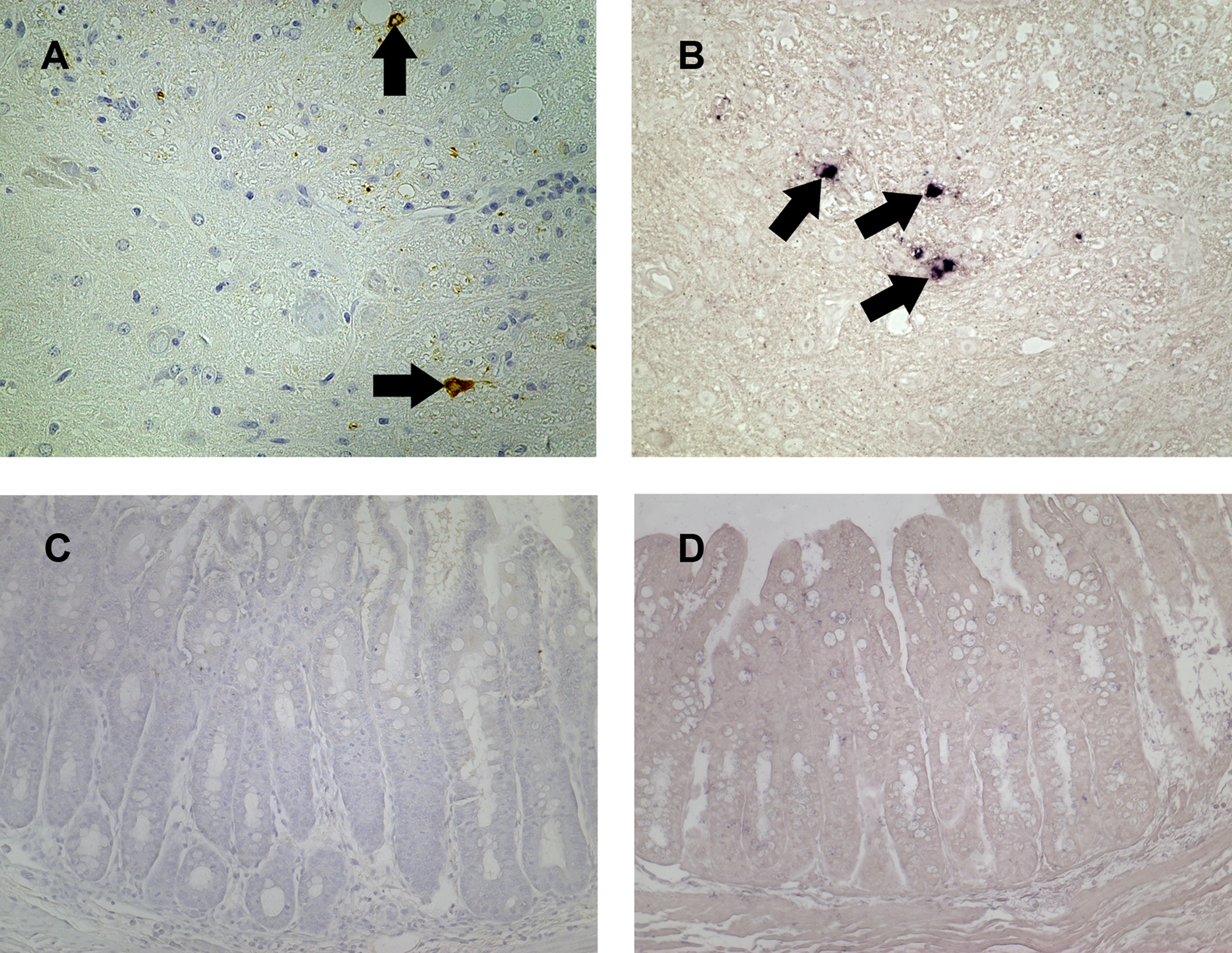

Supplement: S4 Fig — (A) TMEV-specific immunohistochemistry revealed virus antigen (arrows) in the white and adjacent grey matter of the spinal cord at 49 dpi (positive control). (B) Similarly, TMEV-specific in situ hybridization revealed presence of virus RNA in the spinal cord. In contrast, no TMEV-specific antigen (C) or TMEV-specific RNA (D) were detected in the colon of animals. A,C: Immunohistochemistry, 400x, B,D: in situ hybridization, 200x. (TIF) [file pone.0161883.s004.tif]
