# Supplementary material for: Viral Infection of the Central Nervous System Exacerbates Interleukin-10 Receptor Deficiency-Mediated Colitis in SJL Mice
Source: PLoS One. 2016 Sep 9;11(9):e0161883. doi: 10.1371/journal.pone.0161883 (PMC5017624; doi:10.1371/journal.pone.0161883)
Supplement: S1 File — Table A. Summary of statistical analyses of experiment I: effects of IL-10R blockade during acute Theiler’s murine encephalomyelitis. arrows = significant up (↑)- or downregulation (↓) in TMEV-infected SJL mice receiving anti-IL-10R Ab compared with mice receiving IgG1 specific isotype control in the acute phase of the disease; administration of Ab or isotype control, respectively, was performed at 0, 7, 14 and 21 dpi; * bold p-values = significant difference between both groups, p ≤ 0.05, determined by Wilcoxon´s rank sum-tests. TMEV = Theiler´s murine encephalomyelitis virus; dpi = days post infection; IL = interleukin; TNF = tumor necrosis factor; TGF = transmissible growth factor; INF = interferon; Foxp3 = forkhead box P3 protein; MBP = myelin basic protein; np-NF = non-phosphorylated neurofilament; gMFI = geometric mean of fluorescence intensity; ND = not determined. Table B. Summary of statistical analyses of experiment I: effects of acute Theiler’s murine encephalomyelitis virus infection upon IL-10R blockade. arrows = significant up(↑)- or downregulation (↓) in TMEV-infected SJL mice receiving anti-IL-10R Ab compared to non-infected mice receiving anti-IL-10R Ab during the acute infection phase (0, 7, 14 and 21 dpi); * bold p-values display significant difference between both groups (p ≤ 0.05) determined by Wilcoxon´s rank sum-tests. TMEV = Theiler´s murine encephalomyelitis virus; dpi = days post infection; IL = interleukin; TNF = tumor necrosis factor; TGF = transmissible growth factor; INF = interferon; Foxp3 = forkhead box P3 protein; MBP = myelin basic protein; np-NF = non-phosphorylated neurofilament; gMFI = geometric mean of fluorescence intensity; ND = not determined. Table C. Summary of data obtained from animals receiving TMEV-infection and intraperitoneal application of anti-IL-10R antibody (group “IL10R↓early/TMEV” [experiment I] and group “IL10R↓late/TMEV” [experiment II]). TMEV = Theiler´s murine encephalomyelitis virus; dpi = days post infecti [file pone.0161883.s005.doc]

**S1 Table. Statistical analysis and median, minimum and maximum values of performed experiments.**

**Table A. Summary of statistical analyses of *experiment I*: effects of IL-10R blockade during acute Theiler’s murine encephalomyelitis**

|  | **Differences between TMEV-infected SJL mice with (“IL-10R↓_early_/TMEV”) and without (“isotype_early_/TMEV”) IL-10R blockade [p-values]** | | | | |
| --- | --- | --- | --- | --- | --- |
| **Parameter** |  | **7 dpi** | **14 dpi** | **22 dpi** | **42 dpi** |
| **RotaRod®** |  | 0.187 | 0.248 | 0.296 | 0.525 |
| **clinical score** |  | 1.000 | 1.000 | **↑0.007*** | 0.705 |
| **spleen weight** |  | 0.402 | 0.540 | **↑0.012*** | **↑0.006*** |
| **Histology** | leukomyelitis | 1.000 | 0.662 | 0.273 | 0.831 |
| semiquantitative | inflammation small intestine | 1.000 | 1.000 | 1.000 | 1.000 |
| scoring | inflammation cecum | 0.424 | 0.228 | **↑0.040*** | **↑0.001*** |
|  | inflammation colon | 0.239 | **↑0.031*** | **↑0.029*** | **↑0.001*** |
| **Immunohistochemistry** | CD3 | 0.210 | 0.178 | 1.000 | 0.903 |
| spinal cord | CD45R | 0.199 | 0.176 | 0.209 | 0.623 |
|  | Foxp3 | 1.000 | 0.176 | 0.346 | 0.794 |
|  | CD107b | 1.000 | 1.000 | 0.833 | 1.000 |
|  | arginase-1 | 1.000 | 1.000 | 0.737 | 0.714 |
|  | MBP | 1.000 | 0.338 | 1.000 | 0.710 |
|  | np-NF | 1.000 | 0.8413 | 0.270 | 0.976 |
|  | TMEV | 1.000 | 0.194 | 0.728 | 0.461 |
| **RT-PCR** | IL-1α | ND | **↑0.020*** | **↑0.022*** | ND |
| spleen | IL-2 | ND | 0.270 | 0.676 | ND |
| mRNA | IL-4 | ND | 0.178 | 0.095 | ND |
|  | IL-5 | ND | **↑0.018*** | 0.139 | ND |
|  | IL-6 | ND | **↑0.020*** | 0.095 | ND |
|  | TNF | ND | 0.111 | 0.210 | ND |
|  | TGF-β | ND | **↑0.020*** | **↑0.037*** | ND |
|  | IFN-γ | ND | 0.066 | 0.835 | ND |
|  | Foxp3 | ND | 0.066 | 1.000 | ND |
| **RT-PCR** | IL-1α | ND | 0.391 | 0.403 | ND |
| spinal cord | IL-2 | ND | 0.898 | 0.389 | ND |
| mRNA | IL-4 | ND | 1.000 | 0.656 | ND |
|  | IL-5 | ND | 0.540 | 0.666 | ND |
|  | IL-6 | ND | 0.540 | 0.403 | ND |
|  | TNF | ND | 0.540 | 0.676 | ND |
|  | TGF-β | ND | 0.540 | 0.296 | ND |
|  | IFN-γ | ND | 0.806 | 1.000 | ND |
|  | Foxp3 | ND | 0.713 | 0.531 | ND |
|  | TMEV | 0.209 | 0.270 | 1.000 | 0.525 |
| **Flow cytometry** | CD4^+^ | 0.116 | 0.713 | 0.095 | 0.081 |
| spleen | CD8^+^ | 1.000 | 0.391 | 1.000 | 0.079 |
|  | CD19^+^ | 0.144 | 0.325 | **↑0.012*** | 0.081 |
|  | CD4^+^ Foxp3^+^ | 1.000 | 0.903 | **↓0.012*** | 0.081 |
|  | gMFI CD69 gated on CD4^+^ cells | ND | 0.066 | **↑0.012*** | ND |
|  | gMFI CD69 gated on CD8^+^ cells | ND | 0.391 | 0.531 | ND |
|  | gMFI CD44 gated on CD4^+^ cells | ND | **↑0.037*** | 0.210 | ND |
|  | gMFI CD44 gated on CD8^+^ cells | ND | 0.270 | 0.075 | ND |

arrows = significant up (↑)- or downregulation (↓) in TMEV-infected SJL mice receiving anti-IL-10R Ab compared with mice receiving IgG1 specific isotype control in the acute phase of the disease; administration of Ab or isotype control, respectively, was performed at 0, 7, 14 and 21 dpi; ***** bold p-values = significant difference between both groups, p ≤ 0.05, determined by Wilcoxon´s rank sum-tests. TMEV = Theiler´s murine encephalomyelitis virus; dpi = days post infection; IL = interleukin; TNF = tumor necrosis factor; TGF = transmissible growth factor; INF = interferon; Foxp3 = forkhead box P3 protein; MBP = myelin basic protein; np-NF = non-phosphorylated neurofilament; gMFI = geometric mean of fluorescence intensity; ND = not determined.

**Table B. Summary of statistical analyses of *experiment I*: effects of acute Theiler’s murine encephalomyelitis virus infection upon IL-10R blockade**

|  | **Differences between IL-10R blocked SJL mice with (“IL-10R↓_early_/TMEV”) and without (“IL-10R↓_early_/mock”) TMEV-infection [p-values]** | | |
| --- | --- | --- | --- |
| **Parameter** |  | **14 dpi** | **22 dpi** |
| **RotaRod®** |  | 0.307 | 0.762 |
| **clinical score** |  | 1.000 | **↑0.028*** |
| **spleen weight** |  | 0.140 | **↑0.008*** |
| **Histology** | leukomyelitis | **↑0.015*** | **↑0.019*** |
| semiquantitative scoring | inflammation small intestine | 1.000 | 1.000 |
|  | inflammation cecum | 0.380 | 0.749 |
|  | inflammation colon | **↑0.037*** | 0.752 |
| **Immunohistochemistry** | CD3 | **0.016*** | **0.008*** |
| spinal cord | CD45R | **0.008*** | 0.167 |
|  | Foxp3 | **0.008*** | **0.008*** |
|  | CD107b | 1.000 | 0.167 |
|  | arginase-1 | **0.008*** | 0.167 |
|  | MBP | 1.000 | 1.000 |
|  | Np-NF | 0.167 | 0.167 |
| **RT-PCR** | IL-1α | **↑0.020*** | **↑0.008*** |
| spleen | IL-2 | **↑0.020*** | **↑0.008*** |
| mRNA | IL-4 | **↑0.020*** | **↑0.008*** |
|  | IL-5 | **↑0.011*** | **↑0.015*** |
|  | IL-6 | **↑0.020*** | **↑0.008*** |
|  | TNF | **↑0.020*** | **↑0.008*** |
|  | TGF-β | **↑0.020*** | **↑0.008*** |
|  | IFN-γ | **↑0.020*** | **↑0.008*** |
|  | Foxp3 | **↑0.020*** | **↑0.008*** |
| **RT-PCR** | IL-1α | **↑0.020*** | **↑0.036*** |
| spinal cord | IL-2 | 0.308 | **↑0.048*** |
| mRNA | IL-4 | 0.701 | 0.400 |
|  | IL-5 | **↑0.020*** | 0.274 |
|  | IL-6 | **↑0.020*** | 0.083 |
|  | TNF | **↑0.020*** | **↑0.008*** |
|  | TGF-β | **↑0.020*** | 0.235 |
|  | IFN-γ | 0.125 | **↑0.006*** |
|  | Foxp3 | **↑0.020*** | **↑0.036*** |
|  | TMEV | **↑0.011*** | **↑0.004*** |
| **Flow cytometry** | CD4^+^ | **↓0.020*** | ND |
| spleen | CD8^+^ | 0.540 | ND |
|  | CD19^+^ | **↑0.020*** | ND |
|  | CD4^+^ Foxp3^+^ | **↑0.020*** | ND |
|  | gMFI CD69 gated on CD4^+^ cells | **↑0.020*** | ND |
|  | gMFI CD69 gated on CD8^+^ cells | **↑0.020*** | ND |
|  | gMFI CD44 gated on CD4^+^ cells | **↑0.020*** | ND |
|  | gMFI CD44 gated on CD8^+^ cells | **↑0.037*** | ND |

arrows = significant up(↑)- or downregulation (↓) in TMEV-infected SJL mice receiving anti-IL-10R Ab compared to non-infected mice receiving anti-IL-10R Ab during the acute infection phase (0, 7, 14 and 21 dpi); * bold p-values display significant difference between both groups (p ≤ 0.05) determined by Wilcoxon´s rank sum-tests. TMEV = Theiler´s murine encephalomyelitis virus; dpi = days post infection; IL = interleukin; TNF = tumor necrosis factor; TGF = transmissible growth factor; INF = interferon; Foxp3 = forkhead box P3 protein; MBP = myelin basic protein; np-NF = non-phosphorylated neurofilament; gMFI = geometric mean of fluorescence intensity; ND = not determined.

**Table C. Summary of data obtained from animals receiving TMEV-infection and intraperitoneal application of anti-IL-10R antibody (group “IL10R↓_early_/TMEV” [*experiment I*] and group “IL10R↓_late_/TMEV” [*experiment II*])**

| **Parameter** |  | ***Experiment I***  [median value (minimum; maximum)] | | | | ***Experiment II***  [median value (minimum; maximum)] | |
| --- | --- | --- | --- | --- | --- | --- | --- |
|  |  | **7 dpi** | **14 dpi** | **22 dpi** | **42 dpi** | **42 dpi** | **49 dpi** |
| RotaRod®^1^ |  | 13.97 | 8.00 | 14.20 | 9.40 | 9.40 | 7.50 |
|  |  | (12.43; 18.83) | (5.87; 11.53) | (6.47; 19.5) | (6.9; 13.33) | (6.90; 13.33) | (6.00; 9.37) |
| clinical score^2^ |  | 0 | 0 | 1 | 1 | 1 | 1 |
|  |  | (0; 0) | (0; 0) | (1; 3) | (0; 2) | (0; 2) | (0; 4) |
| spleen weight^3^ |  | 0.077 | 0.105 | 0.146 | 0.100 | 0.100 | 0.099 |
|  |  | (0.062; 0.1) | (0.103; 0.114) | (0.142; 0.237) | (0.086; 0.128) | (0.086; 0.128) | (0.081; 0.111) |
| **Histology** | spinal cord^2^ | 0 | 0.125 | 0.167 | 0.542 | 0.583 | 1.333 |
|  |  | (0; 0) | (0.083; 0.167) | (0.083; 0.5) | (0; 1) | (0; 1) | (0,667; 1.417) |
| semiquantitative | small | 0 | 0 | 0 | 0 | 0 | 0 |
| scoring | intestine² | (0; 0) | (0; 0) | (0; 0) | (0; 0) | (0; 0) | (0; 0) |
|  | cecum^2^ | 0 | 1 | 2 | 2 | 0 | 2 |
|  |  | (0; 3) | (0; 5) | (0; 6) | (2; 2) | (0; 3) | (0; 3) |
|  | colon^2^ | 2.5 | 4.5 | 5.2 | 5.0 | 1.0 | 5.2 |
|  |  | (0; 4) | (4; 5) | (3.2; 5.8) | (2.5; 7) | (0; 3.5) | (4.4; 5.6) |
| **IHC** | CD3^4^ | 80.0 | 220.5 | 201.0 | 190.5 | 441.0 | 767.0 |
| spinal cord |  | (33; 178) | (103; 294) | (59; 524) | (9; 972) | (10; 752) | (554; 1122) |
|  | CD45R^4^ | 0 | 11.0 | 4.0 | 17.5 | 190.0 | 215.0 |
|  |  | (0; 2) | (6; 14) | (0; 20) | (0; 78) | (1; 208) | (4; 435) |
|  | Foxp3^4^ | 0 | 17.0 | 15.0 | 32.5 | 60 | 112.0 |
|  |  | (0; 3) | (7; 29) | (1; 28) | (1; 66) | (0; 86) | (56; 147) |
|  | CD107b^4^ | 0 | 0 | 5.0 | 57.0 | 0 | 713.0 |
|  |  | (0; 0) | (0; 0) | (0; 91) | (2; 163) | (0; 334) | (268; 1030) |
|  | arginase-1^4^ | 0 | 0 | 0 | 18.0 | 33 | 279.0 |
|  |  | (0; 0) | (0; 0) | (0; 27) | (0; 103) | (0; 91) | (116; 537) |
|  | MBP^5^ | 0 | 0 | 0 | 0.56 | 4.13 | 6.20 |
|  |  | (0; 0) | (0; 0) | (0; 0.17) | (0; 4.66) | (0; 4.82) | (3.87; 18.54) |
|  | np-NF^6^ | 0 | 0.5 | 8.0 | 113.5 | 226.0 | 272.0 |
|  |  | (0; 0) | (0; 1) | (0; 17) | (3; 260) | (0; 428) | (213; 311) |
|  | TMEV^4^ | 0 | 0.208 | 0.375 | 0.917 | 0.167 | 1.626 |
|  |  | (0; 0) | (0; 0.417) | (0; 1.417) | (0; 2.042) | (0; 2.833) | (1.417; 2.917) |
| **RT-PCR** | IL-1α^7^ | ND | 95136 | 54078 | ND | ND | 136030 |
| **spleen** |  |  | (88853; 123841) | (31999; 111104) |  |  | (61439; 228682) |
| mRNA | IL-2^7^ | ND | 211 | 52 | ND | ND | 194 |
|  |  |  | (88; 313) | (14; 117) |  |  | (60; 377) |
|  | IL-4^7^ | ND | 179 | 86 | ND | ND | 86 |
|  |  |  | (65; 360) | (36; 139) |  |  | (72; 248) |
|  | IL-5^7^ | ND | 104 | 37 | ND | ND | 33 |
|  |  |  | (43; 170) | (0; 68) |  |  | (0; 55) |
|  | IL-6^7^ | ND | 216 | 162 | ND | ND | 314 |
|  |  |  | (164; 338) | (78; 556) |  |  | (132; 412) |
|  | TNF^7^ | ND | 42890 | 13749 | ND | ND | 53474 |
|  |  |  | (25257; 49578) | (7840; 41612) |  |  | (23003; 82110) |
|  | TGF-β^7^ | ND | 699542 | 312326 | ND | ND | 970334 |
|  |  |  | (613117; 805039) | (183678; 1013249) |  |  | (379991; 1377517) |
|  | IFN-γ^7^ | ND | 5328 | 1168 | ND | ND | 5597 |
|  |  |  | (1939; 8415) | (843; 1783) |  |  | (3829; 10809) |
|  | Foxp3^7^ | ND | 766246 | 219181 | ND | ND | 965746 |
|  |  |  | (446524; 955984) | (162410; 457730) |  |  | (531406; 1666715) |

**Table C (continued). Summary of data obtained from animals receiving TMEV-infection and intraperitoneal application of anti-IL-10R antibody (group “IL10R↓_early_/TMEV” [*experiment I*] and group “IL10R↓_late_/TMEV” [*experiment II*])**

| **Parameter** | |  | ***Experiment I***  [median value (minimum; maximum)] | | | | ***Experiment II***  [median value (minimum; maximum)] | |
| --- | --- | --- | --- | --- | --- | --- | --- | --- |
|  | |  | **7 dpi** | **14 dpi** | **22 dpi** | **42 dpi** | **42 dpi** | **49 dpi** |
| **RT-PCR** | IL-1α^7^ | | ND | 5149 | 716 | ND | ND | 15719 |
| **spinal cord** |  | |  | (2300; 5817) | (195; 1347) |  |  | (12944; 50680) |
| mRNA | IL-2^7^ | | ND | 2 | 1 | ND | ND | 10 |
|  |  | |  | (0; 13) | (0; 2) |  |  | (8; 20) |
|  | IL-4^7^ | | ND | 0 | 0 | ND | ND | 4 |
|  |  | |  | (0; 2) | (0; 1) |  |  | (2; 11) |
|  | IL-5^7^ | | ND | 746 | 49 | ND | ND | 105 |
|  |  | |  | (369; 1167) | (0; 122) |  |  | (77; 729) |
|  | IL-6^7^ | | ND | 58 | 7 | ND | ND | 111 |
|  |  | |  | (27; 95) | (1; 37) |  |  | (43; 183) |
|  | TNF^7^ | | ND | 427 | 143 | ND | ND | 3028 |
|  |  | |  | (80; 899) | (77; 203) |  |  | (1599; 6819) |
|  | TGF-β^7^ | | ND | 12422 | 1942 | ND | ND | 17894 |
|  |  | |  | (7479; 21082) | (1208; 3086) |  |  | (9206; 62422) |
|  | IFN-γ^7^ | | ND | 160 | 82 | ND | ND | 2160 |
|  |  | |  | (0; 424) | (32; 325) |  |  | (1209; 7190) |
|  | Foxp3^7^ | | ND | 1577369 | 367484 | ND | ND | 3730999 |
|  |  | |  | (749926; 3338637) | (196170; 584972) |  |  | (1669550; 6942732) |
|  | TMEV^7^ | | 675 | 63166 | 38319 | 272192 | 2765 | 310901 |
|  |  | | (125; 22170) | (302; 336130) | (686; 172860) | (24; 6508670) | (13; 10329892) | (50322; 1932257) |
| **Flow** | CD4^+8^ | | 59.0 | 25.4 | 20.0 | ND | 39.3 | 33.9 |
| **cytometry** |  | | (57.6; 60.8) | (22.8; 26.3) | (14.6; 33.8) |  | (34.8; 42.3) | (30.5; 36.0) |
| spleen | CD8^+8^ | | 23.3 | 8.9 | 12.2 | ND | 13.0 | 13.0 |
|  |  | | (21.3; 27.1) | (8.3; 15.2) | (10.0; 14.9) |  | (11.8; 14.3) | (11.9; 19.9) |
|  | CD19^+8^ | | 42.1 | 55.5 | 34.8 | ND | 37.7 | 40.9 |
|  |  | | (40.2; 44.3) | (48.9; 58.8) | (33.9; 38.7) |  | (34.9; 43.1) | (35.7; 47.0) |
|  | CD4^+^ Foxp3^+8^ | | 4.9 | 10.9 | 2.5 | ND | 9.2 | 3.5 |
|  |  | | (2.3; 10.0) | (4.7; 11.7) | (0.5; 8.3) |  | (8.3; 9.5) | (1.7; 10.8) |
|  | gMFI CD69 (CD4^+^) | | ND | 106 | 115 | ND | ND | 79 |
|  |  | |  | (83; 127) | (73; 137) |  |  | (62; 89) |
|  | gMFI CD69 (CD8^+^) | | ND | 81 | 61 | ND | ND | 61 |
|  |  | |  | (76; 91) | (51; 68) |  |  | (51; 68) |
|  | gMFI CD44 (CD4^+^) | | ND | 2874 | 1915 | ND | ND | 2125 |
|  |  | |  | (2127; 3115) | (1185; 1997) |  |  | (1926; 2253) |
|  | gMFI CD44 (CD8^+^) | | ND | 771 | 365 | ND | ND | 393 |
|  |  | |  | (558; 802) | (340; 457) |  |  | (324; 406) |

TMEV = Theiler´s murine encephalomyelitis virus; dpi = days post infection; IL = interleukin; TNF = tumor necrosis factor; TGF = transmissible growth factor; INF = interferon; Foxp3 = forkhead box P3 protein; MBP = myelin basic protein; np-NF = non-phosphorylated neurofilament; gMFI = geometric mean of fluorescence intensity; ND = not determined; units: ^1^ = rounds per minute; ^2^ = points (semiquantitative scoring); ^3^ = gram; ^4^ = number of labelled cells in the spinal cord; ^5^ = percentage [%] of MBP-unstained (demyelinated) area; ^6^ = number of labelled axons in the spinal cord; ^7^= copy numbers; ^8^ = percentage [%] of labelled cells.

**Table D. Summary of data obtained from animals receiving TMEV-infection and intraperitoneal application of IgG1-specific isotype control (group “isotype_early_/TMEV” [*experiment I*] and group “isotype_late_/TMEV” [*experiment II*])**

| **Parameter** |  | ***Experiment I***  [median value (minimum; maximum)] | | | | | | | | | | ***Experiment II***  [median value (minimum; maximum)] | |
| --- | --- | --- | --- | --- | --- | --- | --- | --- | --- | --- | --- | --- | --- |
|  |  | **7 dpi** | | | | | **14 dpi** | **22 dpi** | | **42 dpi** | | **42 dpi** | **49 dpi** |
| RotaRod®^1^ |  | | | 12.5 | 16.59 | | | | 11.17 | | 6.63 | 6.63 | 8.43 |
|  |  | | | (7.97; 15.23) | (7.30; 22.13) | | | | (9.47; 15.37) | | (6.33; 16.23) | (6.33; 16.23) | (6.67; 10.2) |
| clinical score^2^ |  | | | 0 | 0 | | | | 0 | | 1 | 1 | 1 |
|  |  | | | (0; 0) | (0; 0) | | | | (0; 0) | | (0; 1) | (0; 2) | (1; 1) |
| spleen weight^3^ |  | | | 0.085 | 0.099 | | | | 0.109 | | 0.108 | 0.108 | 0.098 |
|  |  | | | (0.073; 0.089) | (0.06; 0.138) | | | | (0.099; 0.105) | | (0.098; 0.128) | (0.098; 0.128) | (0.085; 0.111) |
| **Histology** | spinal cord^2^ | | | 0 | 0.083 | | | | 0.333 | | 0.666 | 0.916 | 1.000 |
| semiquantitative |  | | | (0; 0) | (0; 0.333) | | | | (0; 0.833) | | (0; 0.833) | (0.166; 1.166) | (0.500; 1.416) |
| scoring | small | | | 0 | 0 | | | | 0 | | 0 | 0 | 0 |
|  | intestine^2^ | | | (0; 0) | (0; 0) | | | | (0; 0) | | (0; 0) | (0; 0) | (0; 0) |
|  | cecum^2^ | | | 0 | 0 | | | | 0 | | 0 | 0 | 0 |
|  |  | | | (0; 0) | (0; 2) | | | | (0; 0) | | (0; 0) | (0; 0) | (0; 0) |
|  | colon^2^ | | | 0 | 0 | | | | 0 | | 0 | 0 | 0 |
|  |  | | | (0; 0) | (0; 0) | | | | (0; 0) | | (0; 0) | (0; 1) | (0; 0) |
| **IHC** | CD3^4^ | | | 40 | 71 | | | | 325 | | 311 | 314 | 393 |
| spinal cord |  | | | (5; 82) | (23; 459) | | | | (18; 514) | | (3; 564) | (225; 686) | (305; 701) |
|  | CD45R^4^ | | | 0 | 1 | | | | 10 | | 22 | 112 | 91 |
|  |  | | | (0; 2) | (0; 50) | | | | (0; 46) | | (0; 85) | (33; 384) | (52; 191) |
|  | Foxp3^4^ | | | 0 | 2 | | | | 29 | | 30 | 24 | 52 |
|  |  | | | (0; 3) | (0; 38) | | | | (1; 41) | | (1; 48) | (2; 94) | (20; 63) |
|  | CD107b^4^ | | | 0 | 0 | | | | 13 | | 25 | 260 | 889 |
|  |  | | | (0; 1) | (0; 0) | | | | (0; 101) | | (0; 124) | (20; 283) | (484; 1388) |
|  | arginase-1^4^ | | | 0 | 0 | | | | 3 | | 42 | 297 | 266 |
|  |  | | | (0; 1) | (0; 0) | | | | (0; 39) | | (0; 73) | (4; 375) | (155; 541) |
|  | MBP^5^ | | | 0 | 0 | | | | 0 | | 0.52 | 1.39 | 12.12 |
|  |  | | | (0; 0) | (0; 0) | | | | (0; 0.22) | | (0; 2.32) | (0.73; 15.29) | (1.58; 15.07) |
|  | np-NF^6^ | | | 0 | 0 | | | | 27 | | 110 | 216 | 225 |
|  |  | | | (0; 1) | (0; 3) | | | | (0; 142) | | (3; 210) | (90; 313) | (58; 296) |
|  | TMEV^4^ | | | 0 | 0 | | | | 0.208 | | 1.208 | 2.500 | 1.916 |
|  |  | | | (0; 0) | (0; 0.083) | | | | (0; 1.083) | | (0; 3.416) | (0.083; 4.417) | (0.500; 2.666) |
| **RT-PCR** | IL-1α^7^ | | | ND | 29903 | | | | 23629 | | ND | ND | 102261 |
| **spleen** |  | | |  | (1662; 68676) | | | | (14721; 43071) | |  |  | (73592; 119636) |
| mRNA | IL-2^7^ | | | ND | 142 | | | | 79 | | ND | ND | 171 |
|  |  | | |  | (1; 160) | | | | (11; 100) | |  |  | (8; 246) |
|  | IL-4^7^ | | | ND | 49 | | | | 59 | | ND | ND | 75 |
|  |  | | |  | (3; 209) | | | | (23; 83) | |  |  | (43; 83) |
|  | IL-5^7^ | | | ND | 0 | | | | 6 | | ND | ND | 48 |
|  |  | | |  | (0; 23) | | | | (0; 29) | |  |  | (18; 100) |
|  | IL-6^7^ | | | ND | 84 | | | | 26 | | ND | ND | 49 |
|  |  | | |  | (6; 129) | | | | (9; 417) | |  |  | (57; 223) |
|  | TNF^7^ | | | ND | 14480 | | | | 9000 | | ND | ND | 38286 |
|  |  | | |  | (553; 38029) | | | | (2771; 19960) | |  |  | (19373; 42174) |
|  | TGF-β^7^ | | | ND | 285394 | | | | 149738 | | ND | ND | 507806 |
|  |  | | |  | (14543; 473350) | | | | (83266; 274446) | |  |  | (414144; 604722) |
|  | IFN-γ^7^ | | | ND | 1784 | | | | 1104 | | ND | ND | 3680 |
|  |  | | |  | | | (26; 2198) | (146; 2009) | |  | |  | (810; 4975) |
|  | Foxp3^7^ | | | ND | 442780 | | | 318192 | | | ND | ND | 881487 |
|  |  | |  | | | (13004; 572834) | | (93258; 514751) | | |  |  | (414934; 1022498) |

**Table D (continued). Summary of data obtained from animals receiving TMEV-infection and intraperitoneal application of IgG1-specific isotype control (group “isotype_early_/TMEV” [*experiment I*] and group “isotype_late_/TMEV” [*experiment II*])**

| **Parameter** | |  | ***Experiment I***  [median value (minimum; maximum)] | | | | ***Experiment II***  [median value (minimum; maximum)] | |
| --- | --- | --- | --- | --- | --- | --- | --- | --- |
|  | |  | **7 dpi** | **14 dpi** | **22 dpi** | **42 dpi** | **42 dpi** | **49 dpi** |
| **RT-PCR** | | IL-1α^7^ | ND | 6052 | 3014 | ND | ND | 27347 |
| **spinal cord** | |  |  | (2521; 7788) | (8; 7095) |  |  | (14141; 71117) |
| mRNA | | IL-2^7^ | ND | 4 | 4 | ND | ND | 18 |
|  | |  |  | (0; 12) | (0; 24) |  |  | (9; 40) |
|  | | IL-4^7^ | ND | 0 | 0 | ND | ND | 3 |
|  | |  |  | (0;1) | (0; 3) |  |  | (0; 26) |
|  | | IL-5^7^ | ND | 551 | 62 | ND | ND | 324 |
|  | |  |  | (185; 1105) | (0; 154) |  |  | (130; 927) |
|  | | IL-6^7^ | ND | 41 | 9 | ND | ND | 241 |
|  | |  |  | (14; 92) | (0; 61) |  |  | (139; 457) |
|  | | TNF^7^ | ND | 549 | 357 | ND | ND | 6959 |
|  | |  |  | (138; 982) | (1; 1259) |  |  | (2920; 156713) |
|  | | TGF-β^7^ | ND | 13946 | 4055 | ND | ND | 32856 |
|  | |  |  | (11370; 25859) | (22; 14143) |  |  | (15312; 129026) |
|  | | IFN-γ^7^ | ND | 199 | 84 | ND | ND | 1679 |
|  | |  |  | (0; 808) | (0; 418) |  |  | (1246; 4902) |
|  | | Foxp3^7^ | ND | 2413158 | 986679 | ND | ND | 4287639 |
|  | |  |  | (910577; 3311236) | (0; 3661683) |  |  | (2239059; 8686385) |
|  | | TMEV^7^ | 70 | 175 | 93605 | 2325294 | 330713 | 339259 |
|  | |  | (0; 13016) | (0; 515721) | (0; 2355452) | (175466; 6001765) | (23089; 1022898) | (41603; 1026877) |
| **Flow** | | CD4^+8^ | 61.1 | 25.3 | 32.3 | ND | 40.6 | 36.2 |
| **cytometry** | |  | (59.0; 63.6) | (24.1; 27.1) | (29.3; 34.9) |  | (34.3; 50.8) | (35.3; 37.8) |
| spleen | | CD8^+8^ | 24.1 | 9.5 | 12.3 | ND | 12.6 | 13.5 |
|  | |  | (22.4; 25.0) | (9.2; 11.6) | (11.4; 14.6) |  | (11.9; 16.5) | (13.1; 14.4) |
|  | | CD19^+8^ | 44.6 | 57.6 | 47.8 | ND | 35.6 | 39.4 |
|  | |  | (40.5; 48.8) | (55.1; 58.8) | (43.3; 51.0) |  | (25.2; 44.7) | (36.0; 44.1) |
|  | | CD4^+^ Foxp3^+8^ | 6.1 | 10.7 | 9.9 | ND | 8.6 | 9.9 |
|  | |  | (3.2; 7.9) | (9.4; 11.4) | (9.1; 10.5) |  | (8.1; 8.9) | (7.4; 11.3) |
|  | | gMFI CD69 (CD4^+^) | ND | 80 | 62 | ND | ND | 76 |
|  | |  |  | (69; 91) | (18; 70) |  |  | (48; 86) |
|  | | gMFI CD69 (CD8^+^) | ND | 88 | 61 | ND | ND | 68 |
|  | |  |  | (77; 95) | (14; 65) |  |  | (48; 76) |
|  | | gMFI CD44 (CD4^+^) | ND | 1882 | 1521 | ND | ND | 1929 |
|  | |  |  | (1853; 2232) | (1459; 1714) |  |  | (1803; 2119) |
|  | | gMFI CD44 (CD8^+^) | ND | 661 | 456 | ND | ND | 380 |
|  |  | |  | (602; 716) | (418; 503) |  |  | (370; 402) |

TMEV = Theiler´s murine encephalomyelitis virus; dpi = days post infection; IL = interleukin; TNF = tumor necrosis factor; TGF = transmissible growth factor; INF = interferon; Foxp3 = forkhead box P3 protein; MBP = myelin basic protein; np-NF = non-phosphorylated neurofilament; gMFI = geometric mean of fluorescence intensity; ND = not determined; units: ^1^ = rounds per minute; ^2^ = points (semiquantitative scoring); ^3^ = gram; ^4^ = number of labelled cells in the cord; ^5^ = percentage [%] of MBP-unstained (demyelinated) area; ^6^ = number of labelled axons in the spinal cord; ^7^= copy numbers; ^8^ = percentage [%] of labelled cells.

**Table E. Summary of data obtained from animals receiving mock-infection and intraperitoneal application of anti-IL-10R antibody (group “isotype_early_/mock” [*experiment I*] and group “isotype_late_/mock” [*experiment II*])**

| **Parameter** |  | ***Experiment I***  [median value (minimum; maximum)] | | ***Experiment II***  [median value (minimum; maximum)] |
| --- | --- | --- | --- | --- |
|  |  | **14 dpi** | **22 dpi** | **49 dpi** |
| **RotaRod®^1^** |  | 15.17 | 13.83 | 15.63 |
|  |  | (7.53; 17.10) | (9.83, 21.27) | (13.1; 19.87) |
| **clinical score ^2^** |  | 0 | 1 | 0 |
|  |  | (0; 0) | (0; 3) | (0; 1) |
| **spleen weight^3^** |  | 0.089 | 0.107 | 0.134 |
|  |  | (0.079; 0.107) | (0.101; 0.141) | (0.117; 0.151) |
| **Histology** | spinal cord^2^ | 0 | 0 | 0 |
| semiquantitative |  | (0; 0) | (0; 0) | (0; 0) |
| scoring | small intestine^2^ | 0 | 0 | 0 |
|  |  | (0; 0) | (0; 0) | (0; 0) |
|  | cecum^2^ | 0 | 2 | 3 |
|  |  | (0; 2) | (0; 3) | (0; 3) |
|  | colon^2^ | 3.2 | 5.4 | 4.6 |
|  |  | (2.4; 4.4) | (1.2; 7.2) | (4.2; 5.0) |
| **IHC** | CD3^4^ | 8 | 4 | 4 |
| spinal cord |  | (3; 13) | (1; 12) | (1; 6) |
|  | CD45R^4^ | 0 | 0 | 0 |
|  |  | (0; 0) | (0; 0) | (0; 0) |
|  | Foxp3^4^ | 0 | 0 | 0 |
|  |  | (0; 0) | (0; 0) | (0; 0) |
|  | CD107b^4^ | 0 | 0 | 0 |
|  |  | (0; 0) | (0; 0) | (0; 0) |
|  | arginase-1^4^ | 0 | 0 | 0 |
|  |  | (0; 0) | (0; 0) | (0; 0) |
|  | MBP^5^ | 0 | 0 | 0 |
|  |  | (0; 0) | (0; 0) | (0; 0) |
|  | np-NF^4^ | 0 | 0 | 0 |
|  |  | (0; 0) | (0; 0) | (0; 0) |
|  | TMEV^4^ | 0 | 0 | 0 |
|  |  | (0; 0) | (0; 0) | (0; 0) |
| **RT-PCR** | IL-1α^6^ | 7066 | 3254 | 8765 |
| **spleen** |  | (3531; 24329) | (2234; 6975) | (2564; 18873) |
| mRNA | IL-2^6^ | 11 | 7 | 16 |
|  |  | (8; 42) | (2; 12) | (10; 30) |
|  | IL-4^6^ | 12 | 7 | 6 |
|  |  | (0; 28) | (2; 16) | (3; 20) |
|  | IL-5^6^ | 0 | 0 | 3 |
|  |  | (0; 0) | (0; 0) | (0; 10) |
|  | IL-6^6^ | 20 | 15 | 32 |
|  |  | (13; 82) | (13; 74) | (9; 58) |
|  | TNF^6^ | 1550 | 660 | 2540 |
|  |  | (620; 3148) | (360; 1699) | (572; 3853) |
|  | TGF-β^6^ | 28098 | 19790 | 52070 |
|  |  | (16316; 103613) | (16055; 46180) | (24148; 79308) |
|  | IFN-γ^6^ | 310 | 135 | 423 |
|  |  | (60; 865) | (97; 196) | (115; 627) |
|  | Foxp3^6^ | 52307 | 34302 | 86165 |
|  |  | (25757; 87927) | (21010; 46309) | (51986; 139657) |

**Table E (continued). Summary of data obtained from animals receiving mock-infection and intraperitoneal application of anti-IL-10R antibody (*experiment I and II*)**

|  |  |  | | | |  |
| --- | --- | --- | --- | --- | --- | --- |
|  |  | | ***Experiment I***  [median value (minimum; maximum)] | | ***Experiment II***  [median value (minimum; maximum)] | |
|  |  | | **14 dpi** | **22 dpi** | **49 dpi** | |
| **RT-PCR** | IL-1α^6^ | | 335 | 176 | 2897 | |
| **spinal cord** |  | | (18; 694) | (52; 422) | (190; 4227) | |
| mRNA | IL-2^6^ | | 0 | 0 | 0 | |
|  |  | | (0; 0) | (0; 0) | (0; 0) | |
|  | IL-4^6^ | | 0 | 0 | 0 | |
|  |  | | (0; 0) | (0; 0) | (0; 0) | |
|  | IL-5^6^ | | 52 | 0 | 187 | |
|  |  | | (0; 114) | (0; 58) | (0; 407) | |
|  | IL-6^6^ | | 4 | 2 | 34 | |
|  |  | | (0; 12) | (1; 4) | (7; 41) | |
|  | TNF^6^ | | 24 | 12 | 126 | |
|  |  | | (0; 41) | (6; 47) | (16; 276) | |
|  | TGF-β^6^ | | 1117 | 744 | 10599 | |
|  |  | | (167; 6372) | (332; 3215) | (1434; 14976) | |
|  | IFN-γ^6^ | | 0 | 0 | 0 | |
|  |  | | (0; 3) | (0; 2.7) | (0; 0) | |
|  | Foxp3^6^ | | 189541 | 154529 | 490640 | |
|  |  | | (11635; 617607) | (0; 331724) | (183215; 1269898) | |
|  | TMEV^6^ | | 0 | 0 | 0 | |
|  |  | | (0; 0) | (0; 0) | (0; 0) | |
| **Flow** | CD4^+7^ | | 31.2 | ND | 30.5 | |
| **cytometry** |  | | (30.4; 37.9) |  | (29.1; 34.4) | |
| spleen | CD8^+7^ | | 9.8 | ND | 8.5 | |
|  |  | | (8.5; 12.4) |  | (7.3; 12.8) | |
|  | CD19^+7^ | | 46.5 | ND | 39.3 | |
|  |  | | (37.3; 48.1) |  | (29.0; 45.8) | |
|  | CD4^+^ Foxp3^+7^ | | 4.1 | ND | 3.2 | |
|  |  | | (1.4; 5.1) |  | (0.7; 7.3) | |
|  | gMFI CD69 (CD4^+^) | | 26.1 | ND | 86 | |
|  |  | | (0; 60.5) |  | (79; 100) | |
|  | gMFI CD69 (CD8^+^) | | 37.5 | ND | 73 | |
|  |  | | (29.5; 40.6) |  | (54; 79) | |
|  | gMFI CD44 (CD4^+^) | | 2181 | ND | 2823 | |
|  |  | | (1993; 2529) |  | (2291; 3251) | |
|  | gMFI CD44 (CD8^+^) | | 442 | ND | 463 | |
|  |  | | (335; 631) |  | (368; 522) | |

TMEV = Theiler´s murine encephalomyelitis virus; dpi = days post infection; IL = interleukin; TNF = tumor necrosis factor; TGF = transmissible growth factor; INF = interferon; Foxp3 = forkhead box P3 protein; MBP = myelin basic protein; np-NF = non-phosphorylated neurofilament; gMFI = geometric mean of fluorescence intensity;ND = not determined; units: ^1^ = rounds per minute; ^2^ = points (semiquantitative scoring); ^3^ = gram; ^4^ = number of labelled cells/axons in the spinal cord; ^5^ = percentage [%] of MBP-unstained (demyelinated) area; ^6^ = copy numbers; ^7^ = percentage [%] of labelled cells

**Table F. Summary of statistical analyses of *experiment II*: effects of IL-10R blockade during chronic Theiler’s murine encephalomyelitis**

|  | **Differences between TMEV-infected SJL mice with (“IL-10R↓_late_/TMEV”) and without (“isotype_late_/TMEV”) IL-10R blockade [p-values]** | | |
| --- | --- | --- | --- |
| **Parameter** |  | **42 dpi** | **49 dpi** |
| **RotaRod®** |  | 0.403 | 0.531 |
| **clinical score** |  | 0.507 | 1.00 |
| **spleen weight** |  | 0.346 | 1.00 |
| **Histology** | leukomyelitis | 0.399 | 0.246 |
| semiquantitative scoring | inflammation small intestine | 1.000 | 1.000 |
|  | inflammation cecum | 0.371 | **↑0.025*** |
|  | inflammation colon | 0.083 | **↑0.007*** |
| **Immunohistochemistry** | CD3 | 0.834 | **↑0.022*** |
| spinal cord | CD45R | 1.000 | 0.210 |
|  | Foxp3 | 0.508 | **↑0.037*** |
|  | CD107b | 0.214 | 0.210 |
|  | arginase-1 | 0.056 | 0.834 |
|  | MBP | 0.508 | 0.167 |
|  | np-NF | 0.944 | 1.000 |
|  | TMEV | 0.249 | 0.531 |
| **RT-PCR** | IL-1α | ND | 0.403 |
| spleen | IL-2 | ND | 0.531 |
| mRNA | IL-4 | ND | 0.210 |
|  | IL-5 | ND | 0.402 |
|  | IL-6 | ND | 0.060 |
|  | TNF | ND | 0.296 |
|  | TGF-β | ND | 0.144 |
|  | IFN-γ | ND | **↑0.037*** |
|  | Foxp3 | ND | 0.403 |
| **RT-PCR** | IL-1α | ND | 0.676 |
| spinal cord | IL-2 | ND | 0.296 |
| mRNA | IL-4 | ND | 0.676 |
|  | IL-5 | ND | 0.144 |
|  | IL-6 | ND | **↓0.037*** |
|  | TNF | ND | 0.144 |
|  | TGF-β | ND | 0.531 |
|  | IFN-γ | ND | 0.834 |
|  | Foxp3 | ND | 1.000 |
|  | MBP | ND | 0.1437 |
|  | TMEV | 0.713 | 1.000 |
| **Flow cytometry** | CD4^+^ | 0.676 | **↓0.037*** |
| spleen | CD8^+^ | 0.917 | 0.296 |
|  | CD19^+^ | 0.531 | 0.402 |
|  | CD4^+^ Foxp3^+^ | 0.144 | 0.095 |
|  | gMFI CD69 gated on CD4^+^ cells | ND | 1.000 |
|  | gMFI CD69 gated on CD8^+^ cells | ND | 0.296 |
|  | gMFI CD44 gated on CD4^+^ cells | ND | 0.095 |
|  | gMFI CD44 gated on CD8^+^ cells | ND | 1.000 |

arrow = significant up(↑)- or downregulation (↓) in TMEV-infected SJL mice receiving anti-IL-10R Ab compared with mice receiving IgG1 specific isotype control in the chronic phase of the disease; administration of Ab or isotype control respectively was performed at 35 and 42 dpi; * bold p-values = significant difference between both groups, p ≤ 0.05, determined by Wilcoxon´s rank sum-tests. TMEV = Theiler´s murine encephalomyelitis virus; dpi = days post infection; IL = interleukin; TNF = tumor necrosis factor; TGF = transmissible growth factor; INF = interferon; Foxp3 = forkhead box P3 protein; MBP = myelin basic protein; np-NF = non-phosphorylated neurofilament; gMFI = geometric mean of fluorescence intensity; ND = not determined.

**Table G. Summary of statistical analyses of *experiment II*: effects of chronic Theiler’s murine encephalomyelitis virus infection upon IL-10R blockade.**

| **Investigation** | **Differences between IL-10R blocked SJL mice with (“IL-10R↓_late_/TMEV”) and without (“IL-10R↓_late_/mock”)**  **TMEV-infection [p-values]** | |
| --- | --- | --- |
|  |  | **49 dpi** |
| **RotaRod®** |  | **↓0.001*** |
| **clinical score** |  | **↑0.003*** |
| **spleen weight** |  | **↓0.012*** |
| **Histology** | leukomyelitis | **↑0.007*** |
| semiquantitative scoring | inflammation small intestine | 1.000 |
|  | inflammation cecum | 0.515 |
|  | inflammation colon | 0.432 |
| **Immunohistochemistry** | CD3 | **0.008*** |
| spinal cord | CD45R | **0.008*** |
|  | Foxp3 | **0.008*** |
|  | CD107b | **0.008*** |
|  | Arginase-1 | **0.008*** |
|  | MBP | **0.008*** |
|  | np-NF | **0.008*** |
| **RT-PCR** | IL-1α | **↑0.012*** |
| spleen | IL-2 | **↑0.012*** |
| mRNA | IL-4 | **↑0.012*** |
|  | IL-5 | 0.389 |
|  | IL-6 | **↑0.012*** |
|  | TNF | **↑0.012*** |
|  | TGF-β | **↑0.012*** |
|  | IFN-γ | **↑0.012*** |
|  | Foxp3 | **↑0.012*** |
| **RT-PCR** | IL-1α | **↑0.012*** |
| spinal cord | IL-2 | **↑0.008*** |
| mRNA | IL-4 | **↑0.008*** |
|  | IL-5 | 0.676 |
|  | IL-6 | **↑0.012*** |
|  | TNF | **↑0.012*** |
|  | TGF-β | 0.060 |
|  | IFN-γ | **↑0.008*** |
|  | Foxp3 | **↑0.012*** |
|  | TMEV | **↑0.008*** |
| **Flow cytometry** | CD4^+^ | 0.296 |
| spleen | CD8^+^ | **↑0.037*** |
|  | CD19^+^ | 0.296 |
|  | CD4^+^ Foxp3^+^ | 0.531 |
|  | gMFI CD69 gated on CD4^+^ cells | 0.403 |
|  | gMFI CD69 gated on CD8^+^ cells | 0.144 |
|  | gMFI CD44 gated on CD4^+^ cells | 0.210 |
|  | gMFI CD44 gated on CD8^+^ cells | 0.095 |

arrows = significant up-regulation (↑) or down-regulation (↓) in infected SJL mice receiving IL-10R Ab compared to non-infected mice receiving anti-IL-10R Ab during the chronic infection phase (35 and 42 dpi); * bold p-values display significant differences between both groups (p ≤ 0.05) determined by Wilcoxon´s rank sum-tests. TMEV = Theiler´s murine encephalomyelitis virus; dpi = days post infection; Foxp3 = forkhead box P3 protein; MBP = myelin basic protein; np-NF = non-phosphorylated neurofilament; gMFI = geometric mean of fluorescence intensity.

**Table H. Summary of statistical analyses of *experiment III*: effects of IL-10R blockade in non-infected animals**

|  | **Differences between IL-10R blocked SJL mice (“IL-10R↓”) and**  **non-treated animals (“isotype”) [p-values]** | | | |
| --- | --- | --- | --- | --- |
| **Parameter** |  | **day 7** | **day 14** | **day 21** |
| **Histology** | inflammation small intestine | 1.000 | 1.000 | 1.000 |
| semiquantitative scoring | inflammation cecum | 1.000 | 0.067 | **↑0.007*** |
|  | inflammation colon | 0.067 | **↑0.007*** | **↑0.006*** |
| **Immunohistochemistry** | CD3 | **↑0.024*** | **↑0.008*** | **↑0.008*** |
| colon | CD45R | 0.580 | 0.064 | **↑0.008*** |
|  | Foxp3 | **↑0.040*** | **↑0.008*** | **↑0.008*** |
|  | Iba-1 | **↑0.008*** | **↑0.008*** | **↑0.008*** |
| **Flow cytometry** | CD3^+^ | 1.000 | 0.402 | 0.143 |
| spleen | CD4^+^ | 1.000 | 0.463 | 0.835 |
|  | CD8^+^ | 1.000 | 0.403 | 0.210 |
|  | CD19^+^ | 0.403 | 1.000 | 0.060 |
|  | CD4^+^ Foxp3^+^ | 1.000 | 0.676 | **↓0.021*** |
|  | gMFI CD69 gated on CD4^+^ cells | 0.530 | 1.000 | **↑0.012*** |
|  | gMFI CD69 gated on CD8^+^ cells | 1.000 | 1.000 | **↑0.012*** |
|  | gMFI CD44 gated on CD4^+^ cells | 0.676 | 0.531 | 0.144 |
|  | gMFI CD44 gated on CD8^+^ cells | 0.835 | 0.403 | **↑0.012*** |

arrow = significant up(↑)- or downregulation (↓) in SJL mice receiving anti-IL-10R Ab compared with mice receiving IgG1 specific isotype only; administration of Ab or isotype control ,respectively, was performed at day 0, 7 and 14; * bold p-values = significant difference between both groups, p ≤ 0.05, determined by Wilcoxon´s rank sum-tests. Foxp3 = Iba-1 = ionized calcium binding adaptor molecule 1; forkhead box P3 protein; gMFI = geometric mean of fluorescence intensity.

**Table I. Summary of data obtained from non-infected animals receiving intraperitoneal application of anti-IL10R Ab (group “IL10R↓”; *experiment III*)**

| **Parameter** |  | | **day 7*** | **day 14*** | **day 21*** |
| --- | --- | --- | --- | --- | --- |
| **Histology** | | small intestine^1^ | 0 | 0 | 0 |
| semiquantitative | |  | (0; 0) | (0; 0) | (0; 0) |
| scoring | | cecum^1^ | 0 | 2 | 2 |
|  | |  | (0; 0) | (0; 2) | (2; 3) |
|  | | colon^1^ | 2 | 4 | 8 |
|  | |  | (0; 2) | (2; 5) | (7; 8) |
| **Immunohistochemistry** | | CD3^2^ | 33.6 | 48.2 | 47.2 |
| colon | |  | (24.4; 54.0) | (36.6; 56.0) | (38.6; 58.6) |
|  | | CD45R^2^ | 5.0 | 13.0 | 13.4 |
|  | |  | (3.6; 6.0) | (3.0; 24.2) | (9.8; 15.4) |
|  | | Foxp3^2^ | 3.2 | 9.9 | 10.8 |
|  | |  | (1.0; 10.5) | (2.0; 16.7) | (7.7; 14.1) |
|  | | Iba-1^2^ | 32.3 | 48.8 | 60.4 |
|  | |  | (18.6; 48.7) | (44.4; 75.0) | (44.2; 65.6) |
| **Flow cytometry** | | CD3^+3^ | 46.4 | 46.7 | 54.8 |
| spleen | |  | (44.0; 54.5) | (45.6; 51.8) | (49.8; 58.2) |
|  | | CD4^+3^ | 70.5 | 72.2 | 70.5 |
|  | |  | (68.8; 72.4) | (72.0; 73.1) | (68.9; 72.3) |
|  | | CD8^+3^ | 26.2 | 24.9 | 26.6 |
|  | |  | (25.1; 28.0) | (23.9; 25.4) | (25.0; 28.2) |
|  | | CD19^+3^ | 48.0 | 40.6 | 37.4 |
|  | |  | (41.5; 50.0) | (35.0; 45.6) | (35.8; 43.5) |
|  | | CD4^+^ Foxp3^+3^ | 7.7 | 7.6 | 6.7 |
|  | |  | (6.0; 8.1) | (6.9; 8.8) | (6.4; 7.0) |
|  | | gMFI CD69 (CD4^+^) | 52 | 118 | 45.3 |
|  | |  | (47; 53) | (116; 135) | (42.0; 49.6) |
|  | | gMFI CD69 (CD8^+^) | 59 | 114 | 38.3 |
|  | |  | (54; 62) | (111; 131) | (35.7; 41.6) |
|  | | gMFI CD44 (CD4^+^) | 649 | 626 | 568 |
|  | |  | (625; 733) | (501; 734) | (539; 707) |
|  | | gMFI CD44 (CD8^+^) | 270 | 233 | 179 |
|  | |  | (253; 323) | (192; 270) | (172; 197) |

* given are median values (minimum and maximum); gMFI = geometric mean of fluorescence intensity; units: ^1^ = points (semiquantitative scoring); ^2^ = number of labelled cells in the *lamina propria* per high power field, ^3^ = percentage [%] of labelled cells

**Table J. Summary of data obtained from non-infected animals receiving intraperitoneal application of IgG1-specific isotype control (group “isotype”; *experiment III*)**

|  |  | |  | | | |
| --- | --- | --- | --- | --- | --- | --- |
| **Parameter** |  | | | **day 7*** | **day 14*** | **day 21*** |
| **Histology** | | small intestine^1^ | | 0 | 0 | 0 |
| semiquantitative | |  | | (0; 0) | (0; 0) | (0; 0) |
| scoring | | cecum^1^ | | 0 | 0 | 0 |
|  | |  | | (0; 0) | (0; 0) | (0; 0) |
|  | | colon^1^ | | 0 | 0 | 0 |
|  | |  | | (0; 0) | (0; 0) | (0; 0) |
| **Immunohistochemistry** | | CD3^2^ | | 21.8 | 15.6 | 14.0 |
| colon | |  | | (13.0; 26.4) | (14.6; 19.4) | (11.6; 17.6) |
|  | | CD45R^2^ | | 4.6 | 3.2 | 3.6 |
|  | |  | | (1.6; 5.6) | (1.0; 4.0) | (2.8; 4.8) |
|  | | Foxp3^2^ | | 1.0 | 0.9 | 1.2 |
|  | |  | | (0.2; 1.2) | (0.4; 1.4) | (0.5; 2.1) |
|  | | Iba-1^2^ | | 16.6 | 18.0 | 17.4 |
|  | |  | | (11.6; 17.4) | (12.8; 25.4) | (14.2; 22.6) |
| **Flow cytometry** | | CD3^+3^ | | 48.3 | 44.7 | 49.4 |
| spleen | |  | | (39.5; 51.4) | (43.2; 50.2) | (40.0; 59.3) |
|  | | CD4^+3^ | | 70.7 | 71.0 | 72.0 |
|  | |  | | (68.0; 72.7) | (69.7; 73.2) | (61.7; 74.2) |
|  | | CD8^+3^ | | 25.9 | 25.6 | 24.7 |
|  | |  | | (24.6; 28.0) | (24.0; 27.1) | (21.4; 35.1) |
|  | | CD19^+3^ | | 44.0 | 42.9 | 31.6 |
|  | |  | | (39.5; 52.5) | (28.6; 47.0) | (25.4; 39.2) |
|  | | CD4^+^ Foxp3^+3^ | | 7.9 | 6.7 | 7.3 |
|  | |  | | (1.4; 10.2) | (5.0; 9.7) | (6.9; 9.8) |
|  | | gMFI CD69 (CD4^+^) | | 49 | 124 | 38.5 |
|  | |  | | (48; 52) | (102; 134) | (34.5; 40.9) |
|  | | gMFI CD69 (CD8^+^) | | 58 | 120 | 38.3 |
|  | |  | | (56; 61) | (99; 128) | (35.7; 41.6) |
|  | | gMFI CD44 (CD4^+^) | | (682 | 570 | 488 |
|  | |  | | (619; 735) | (524; 745) | (462; 720) |
|  | | gMFI CD44 (CD8^+^) | | 278 | 212 | 179 |
|  | |  | | (251; 307) | (175; 256) | (172; 197) |

* given are median values (minimum and maximum); gMFI = geometric mean of fluorescence intensity; units: ^1^ = points (semiquantitative scoring); ^2^ = number of labelled cells in the *lamina propria* per high power field; ^3^ = percentage [%] of labelled cells
